# Supplementary material for: Predicted functional consequences of WNT ligand mutations in colorectal cancer
Source: Biophys J. 2025 Mar 31;124(9):1496–505. doi: 10.1016/j.bpj.2025.03.030 (PMC12256883; doi:10.1016/j.bpj.2025.03.030)
Supplement: Document S1. Figures S1–S8 [file mmc1.pdf]

Biophysical Journal, Volume *124*

Supplemental information

Predicted functional consequences of WNT ligand mutations in color-ectal cancer

Aamir Ahmed and David Shorthouse

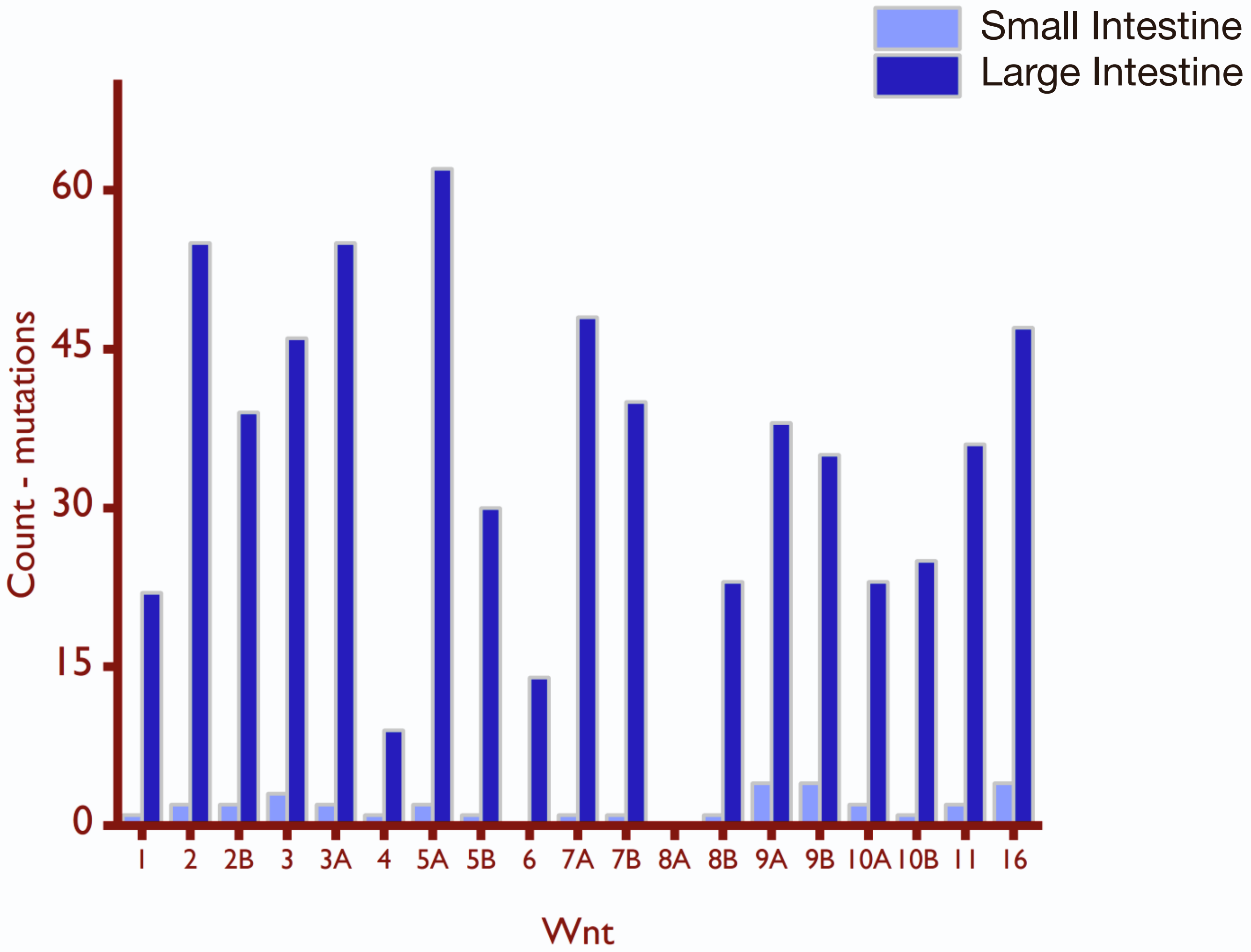

Figure S1: Mutational frequency of human Wnt genes in human cancers of the Large Intestine (dark blue), and Small Intestine (light blue). Data taken from COSMIC.

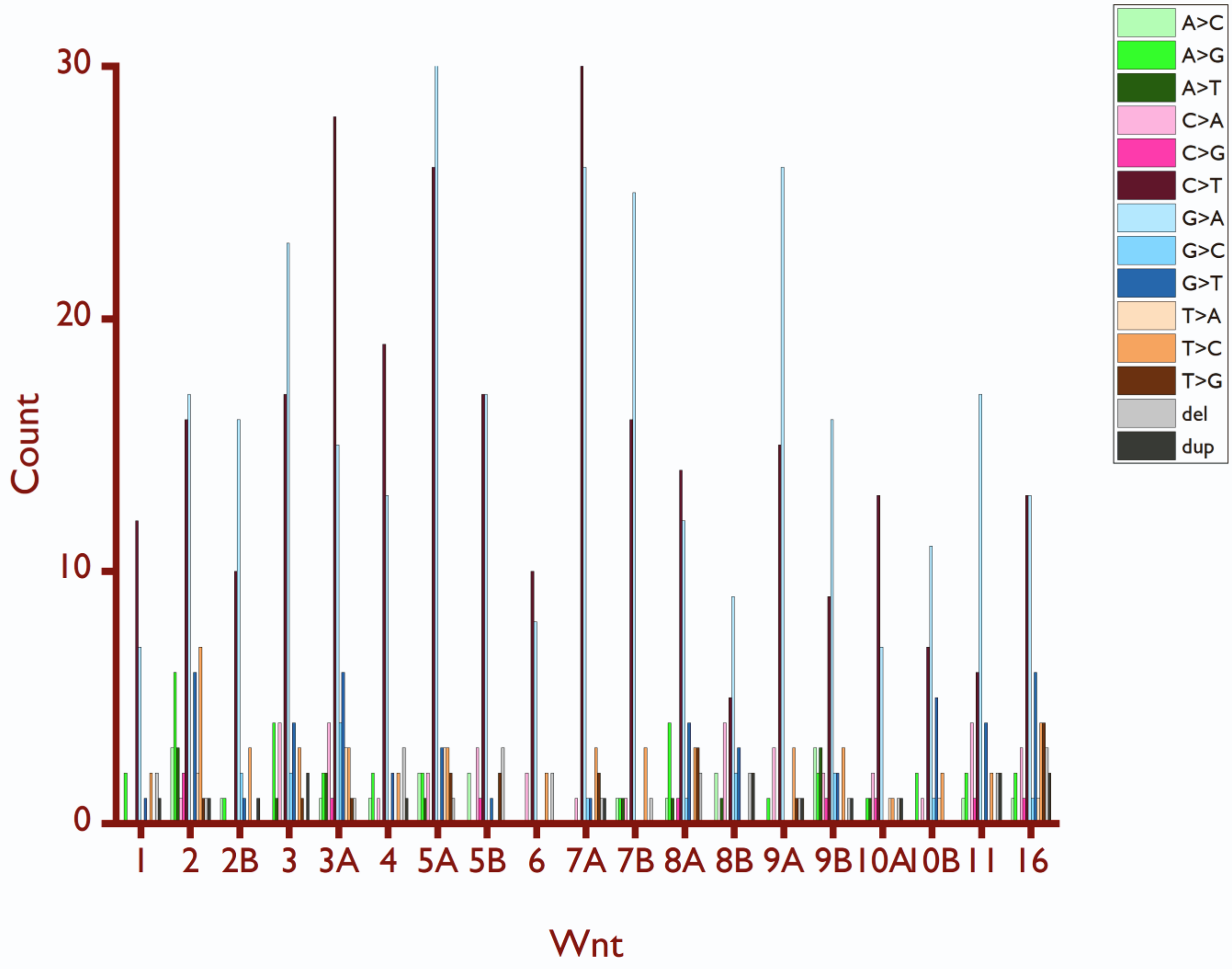

Figure S2: Mutational signatures found in Wnt genes in cancers of the Large Intestine. Data taken from COSMIC.

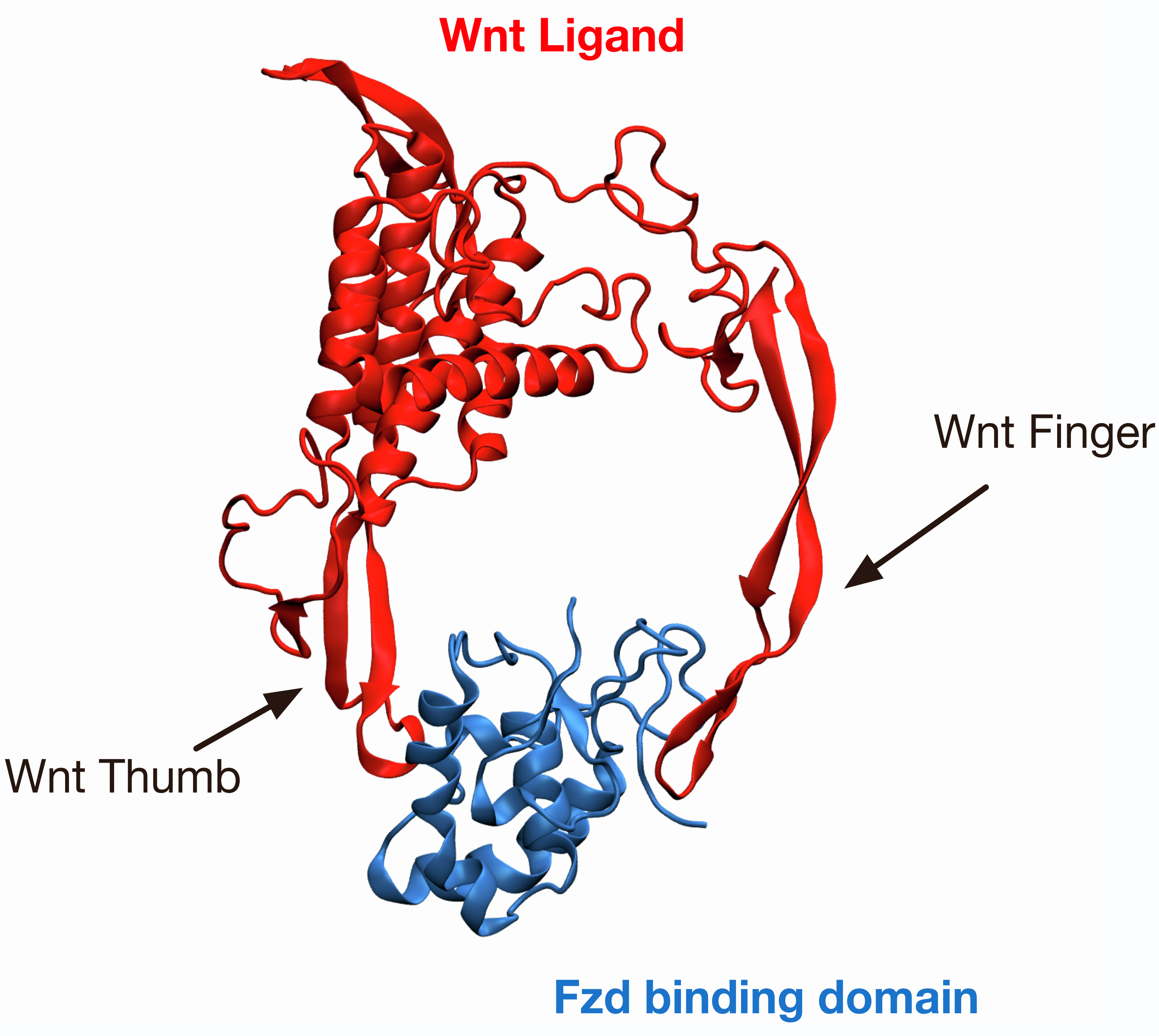

Figure S3: Structure of the Wnt/Fzd interaction, highlighting the Thumb and Finger domains.

```
Length: 696
Identity:     664/696 (95.4%)
Similarity:   667/696 (95.8%)
Gaps:          13/696 ( 1.9%)
```

- Covered in Crystal Structure 6AHY

```
sp|Q9H461|FZD8_HUMAN      MEWGYLLEVTSLLAALALLQRSSGAAAASAKELACQEITVPLCKGIGYNYTYMPNQFNHD   60
sp|Q61091|FZD8_MOUSE      MEWGYLLEVTSLLAALAVLQRSSGAAAASAKELACQEITVPLCKGIGYNYTYMPNQFNHD   60
                          *****************:******************************************

sp|Q9H461|FZD8_HUMAN      TQDEAGLEVHQFWPLVEIQCSPDLKFFLCSMYTPICLEDYKKPLPPCRSVCERAKAGCAP   120
sp|Q61091|FZD8_MOUSE      TQDEAGLEVHQFWPLVEIQCSPDLKFFLCSMYTPICLEDYKKPLPPCRSVCERAKAGCAP   120
                          ************************************************************

sp|Q9H461|FZD8_HUMAN      LMRQYGFAWPDRMRCDRLPEQGNPDTLCMDYNRTDLTTAAPSPPRRLPP-PPPGEQPPSG   179
sp|Q61091|FZD8_MOUSE      LMRQYGFAWPDRMRCDRLPEQGNPDTLCMDYNRTDLTTAAPSPPRRLPPPPPPGEQPPSG   180
                          ************************************************* **********

sp|Q9H461|FZD8_HUMAN      SGHGRPPGARPPHRGGGRGGGGGDAAAPPARGGGGGGKARPPGGGAAPCEPGCQCRAPMV   239
sp|Q61091|FZD8_MOUSE      SGHSRPPGARPPHRGGSSRGSGDAAAAPPSRG----GKARPPGGGAAPCEPGCQCRAPMV  236
                          ***.************.  *.*. *****:**    ************************

sp|Q9H461|FZD8_HUMAN      SVSSERHPLYNRVKTGQIANCALPCHNPFFSQDERAFTVFWIGLWSVLCFVSTFATVSTF   299
sp|Q61091|FZD8_MOUSE      SVSSERHPLYNRVKTGQIANCALPCHNPFFSQDERAFTVFWIGLWSVLCFVSTFATVSTF   296
                          ************************************************************

sp|Q9H461|FZD8_HUMAN      LIDMERFKYPERPIIFLSACYLFVSVGYLVRLVAGHEKVACSGGAPGAGGAGGAGGA-AA   358
sp|Q61091|FZD8_MOUSE      LIDMERFKYPERPIIFLSACYLFVSVGYLVRLVAGHEKVACSGGAPGAGGAGGAGGAAAA   356
                          ********************************************************* **

sp|Q9H461|FZD8_HUMAN      GAGAAGAGAGGPGGRGEYEELGAVEQHVRYETTGPALCTVVFLLVYFFGMASSIWWVILS   418
sp|Q61091|FZD8_MOUSE      GAGAAGAGASSPGARGEYEELGAVEQHVRYETTGPALCTVVFLLVYFFGMASSIWWVILS   416
                          *********..**.**********************************************

sp|Q9H461|FZD8_HUMAN      LTWFLAAGMKWGNEAIAGYSQYFHLAAWLVPSVKSIAVLALSSVDGDPVAGICYVGNQSL   478
sp|Q61091|FZD8_MOUSE      LTWFLAAGMKWGNEAIAGYSQYFHLAAWLVPSVKSIAVLALSSVDGDPVAGICYVGNQSL   476
                          ************************************************************

sp|Q9H461|FZD8_HUMAN      DNLRGFVLAPLVIYLFIGTMFLLAGFVSLFRIRSVIKQQDGPTKTHKLEKLMIRLGLFTV   538
sp|Q61091|FZD8_MOUSE      DNLRGFVLAPLVIYLFIGTMFLLAGFVSLFRIRSVIKQQGGPTKTHKLEKLMIRLGLFTV   536
                          ***************************************.********************

sp|Q9H461|FZD8_HUMAN      LYTVPAAVVVACLFYEQHNRPRWEATHNCPCLRDLQPDQARRPDYAVFMLKYFMCLVVGI   598
sp|Q61091|FZD8_MOUSE      LYTVPAAVVVACLFYEQHNRPRWEATHNCPCLRDLQPDQARRPDYAVFMLKYFMCLVVGI   596
                          ************************************************************

sp|Q9H461|FZD8_HUMAN      TSGVWVWSGKTLESWRSLCTRCCWASKGAAVGGGAGATAAGGGGGPGGGGGGGPGGGGGP   658
sp|Q61091|FZD8_MOUSE      TSGVWVWSGKTLESWRALCTRCCWASKGAAVGAGAGGSG-------PGGSGPGPGGGGGH 649
                          ****************:***************.***.:.       **.* *******

sp|Q9H461|FZD8_HUMAN      GGGGGSLYSDVSTGLTWRSGTASSVSYPKQMPLSQV 694
sp|Q61091|FZD8_MOUSE      GGGGGSLYSDVSTGLTWRSGTASSVSYPKQMPLSQV 685
                          ************************************
```

Figure S4: Sequence alignment of human and murine Fzd8.

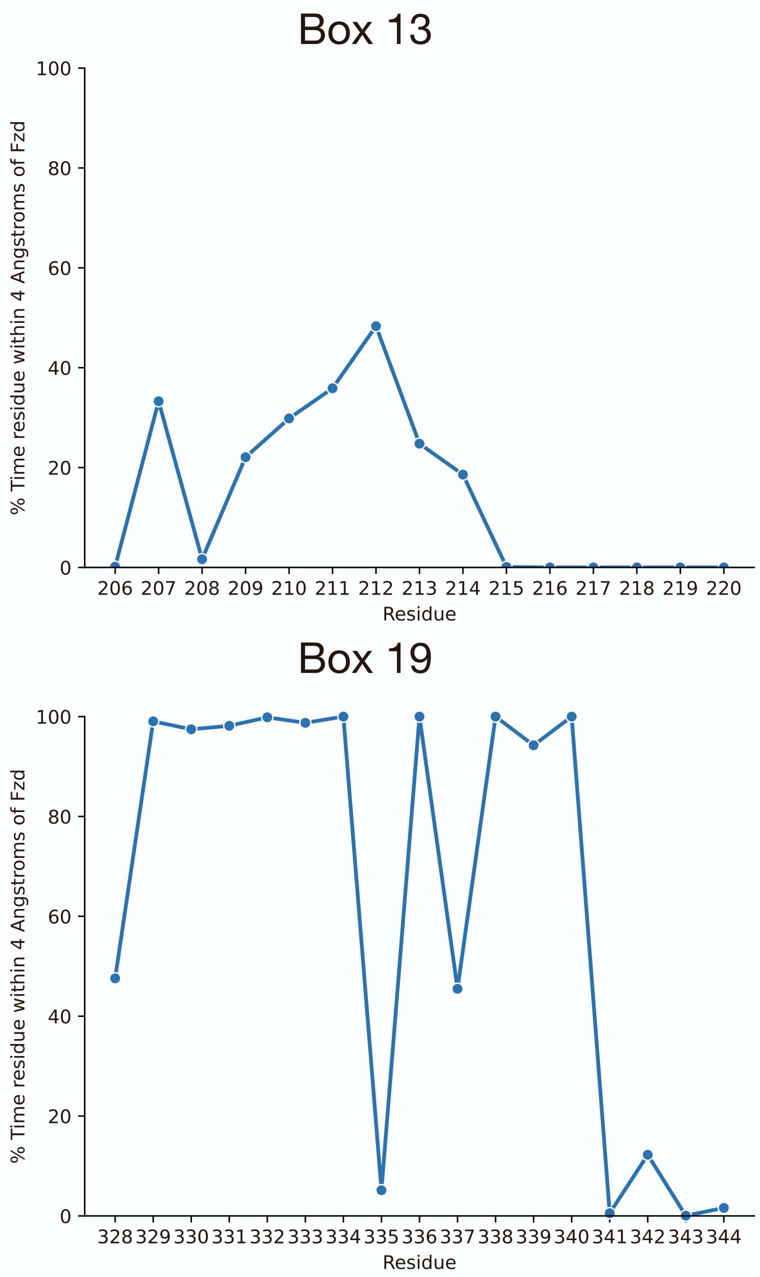

Figure S5: Residence time of the interaction of each amino acid in box 13 and 19 with the Fzd protein. A residue is determined to be in contact with Fzd if it is within 4 Angstroms of any atom in a Fzd residue.

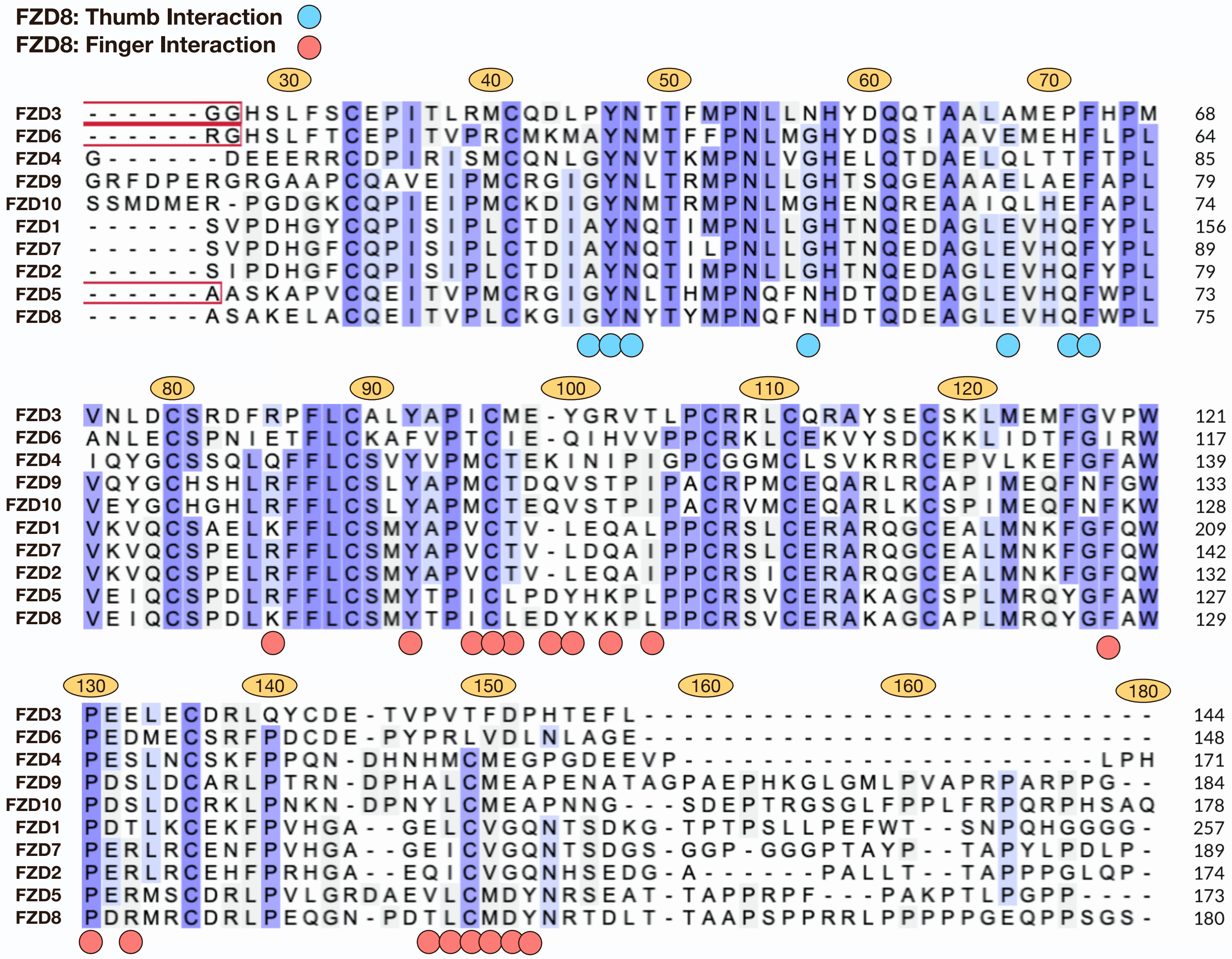

Figure S6: Sequence alignment of all 10 human Fzd protein sequences in the regions interacting with Wnt ligands. Residues interacting (Defined as within 5 angstroms of the Wnt ligand) are highlighted in Blue (Thumb interaction), and Red (Finger interaction).

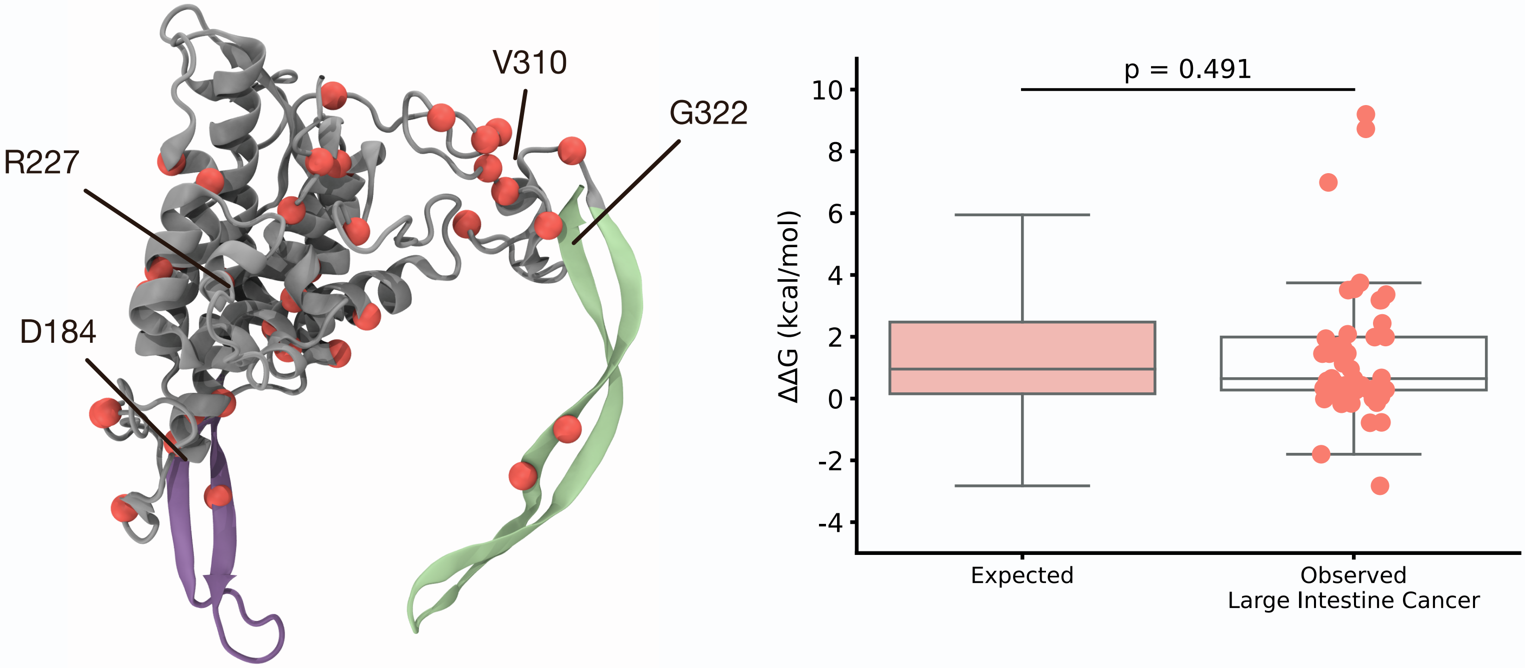

Figure S7. Mutations to Wnt5a found in patients with colorectal adenocarcinoma. (Left) Structure of Wnt5a with mutation sites highlighted as red spheres. (Right) Comparison of folding energy of each mutation (kcal/mol) compared to an expected distribution calculated from the mutational signature. P represents monte-carlo test.

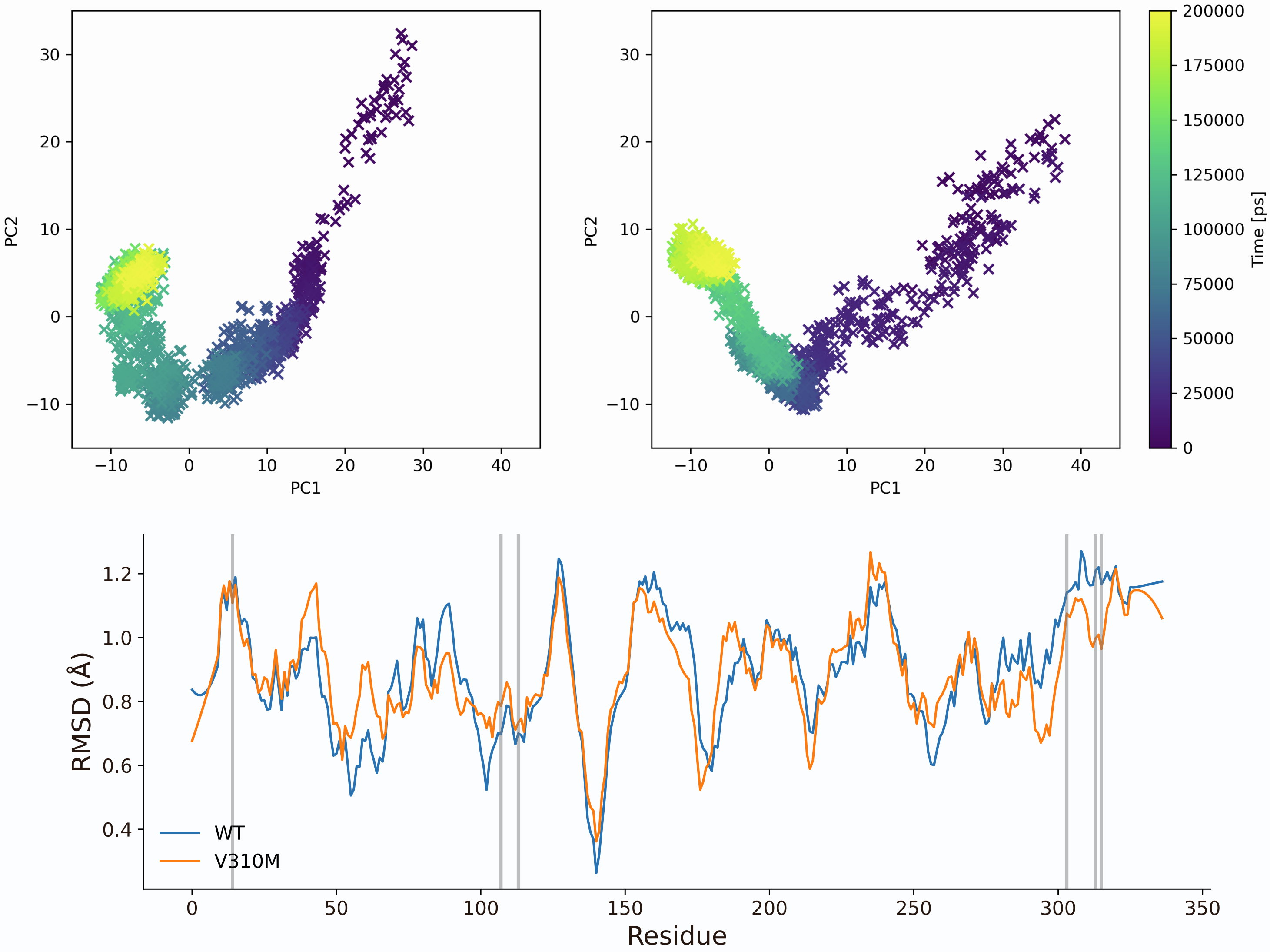

Figure S8: Principal Component Analysis (PCA) for 200ns molecular dynamics simulations for: (top left) WT human WNT5A, (top right) V310M mutated human WNT5A. (Bottom) Root mean square deviation (RMSD) calculations for the trajectories, hinge residues are highlighted as grey lines.
