# Supplementary material for: Predicted functional consequences of WNT ligand mutations in colorectal cancer
Source: Biophys J. 2025 Mar 31;124(9):1496–505. doi: 10.1016/j.bpj.2025.03.030 (PMC12256883; doi:10.1016/j.bpj.2025.03.030)
Supplement: Document S2. Article plus supporting material [file mmc2.pdf]

Biophysical Journal
Article

Biophysical Society

Predicted functional consequences of WNT ligand mutations in colorectal cancer

Aamir Ahmed[1,*] and David Shorthouse[2,*]
[1]Cell and Developmental Biology, University College London, London, United Kingdom and [2]School of Pharmacy, University College London, London, United Kingdom

ABSTRACT Mutations to wingless integration site (WNT) ligands in cancer are poorly understood. WNT ligands are a family of secreted proteins that trigger the activation of the WNT pathway, with essential roles in cell development and carcinogenesis, particularly in the colorectal tract. While the structure of WNT ligands has been elucidated, little is known about how mutations in these proteins affect colorectal cancer. Here, we show that mutations in WNT ligands found in colorectal cancer show regional specificity and selectivity for particular conserved sequences. We further show that mutations in colorectal cancer are not selecting for changes in the binding affinity of the ligands to their receptor. We use clinical data to identify mutations to WNT5A as under selection and correlating with patient outcomes in colorectal cancer, and by combining mutational data and folding energy calculations, elastic network modeling, and molecular dynamics simulations, we show that these mutations alter its structural dynamics and flexibility. Thus, we predict a novel structure-function relationship for mutations in WNT ligands in human cancers.

SIGNIFICANCE Wingless integration site (WNT) signaling plays an essential role in cancer development—particularly for colorectal adenocarcinoma. Despite this importance, very little is known about the functional impact of mutations in WNT ligands. Here, we perform a detailed analysis of mutations to WNT ligands in colorectal adenocarcinoma. We combine primary sequence analysis, saturation folding energy calculation, and statistical analysis to assess the structural and functional roles of mutations in these proteins. We demonstrate that WNT5A mutations correlate with outcomes in colorectal cancer and find that, contrary to expectations, mutations do not impact WNT ligand binding to their respective receptors but instead appear to influence the dynamics and flexibility of the proteins. We confirm this hypothesis with molecular dynamics simulations.

INTRODUCTION

Wingless integration site (WNT) signaling plays an essential role in numerous critical cellular processes, including embryogenesis, during development, in the maintenance of homeostasis, and in a variety of diseases, including carcinogenesis (1). WNT signaling initiates when WNTs, soluble secreted proteins that act as paracrine signaling molecules, bind to Frizzled (FZD) receptors and/or activate ion channels on mammalian cell membranes (2). The WNT signal is transduced through two major intracellular transducers, triggering the release of WNT-induced free intracellular calcium (3) and stabilization of the transcription factor coactivator β-catenin, a 92 kDa protein (4). Stabilized β-catenin enters the nucleus, a process facilitated by the simultaneous availability of intracellular calcium (5), and activates gene transcription; many of the genes under the WNT/β-catenin transcriptional control are proto-oncogenes (4).

There are 19 human WNTs (e.g., WNT, -3A, -5A, -9B, -10B, and -11) and 10 human FZD genes, in addition to nine LRP coreceptors (1). It is possible that this redundancy is an evolutionary byproduct of the importance of WNT signaling due to their early evolution and presence across biology from protists to humans (6). Mutations in the genes encoding for various components of WNT signaling pathways are thought to play a major role in carcinogenesis, immune evasion by cancer cells (7), and metastasis (8). For example, mutations in the APC gene, which encodes for a member of the so-called destruction complex, responsible for preventing β-catenin from being stabilized, is found to be mutated in 48% of large intestine and 22% of small intestine cancers (Database COSMIC: https://cancer.sanger.ac.

Submitted September 23, 2024, and accepted for publication March 26, 2025.

*Correspondence: aamir.ahmed@ucl.ac.uk or d.shorthouse@ucl.ac.uk

Editor: Abhishek Singharoy.
https://doi.org/10.1016/j.bpj.2025.03.030

© 2025 The Author(s). Published by Elsevier Inc. on behalf of Biophysical Society.
This is an open access article under the CC BY license (http://creativecommons.org/licenses/by/4.0/).

uk/cosmic, retrieved February 24, 2024). Similarly, WNT ligand genes are mutated in 13.6% and 6.3% of large intestine and small intestine cancers, respectively. Indeed, mutations in almost all components of WNT signaling pathway genes have been identified (COSMIC).

Despite the importance of WNT signaling in colorectal (and other) cancers, a detailed analysis of primary and tertiary structures alongside mutations in WNT ligand genes and how they might alter structure has been lacking. There is little information regarding the structure-function relationships and biophysical properties of WNTs and FZD receptors performed in a systematic manner other than a study by Agostino et al. (9), who used homology modeling for a series of WNT/FZD interactions and determined the predicted binding affinities of WNT/FZD pairs using a quantitative structure-activity relationship model. Of the human FZD receptors, FZD4 has a crystal structure resolved at 2.4 Å in a ligand-free state (10), and murine FZD8 (which shares 95.4% sequence identity to human and 100% identity in the region that binds to WNT) has been structurally characterized to be bound to the human WNT3 ligand (11). Our motivation for this study was driven by an earlier analysis of the primary sequence of human WNT proteins, which showed key conserved motifs between WNT11 and other WNTs (such as WNT3A, -4, -5A, -7A, -8, -9B, and -10B) (5). Since then, the availability of tools such as AlphaFold (12) has allowed the modeling of protein structures that can be used alongside statistical tools (13–15) to study mutations and their potential functional consequences, as reviewed in (16). Data from such analyses can be used to generate hypothesis-led experiments to further understand the function of key signaling pathways in diseases such as cancers. While there has been some analysis of the WNT ligand structure previously (9,17), these reports have not investigated the consequences of mutations observed in cancer, instead studying properties of the WNT-FZD signaling interaction.

We have used primary sequence analysis and structural modeling coupled with energy calculations, along with statistical analysis techniques, to study the structures and mutations in human WNTs in colorectal cancer.

MATERIALS AND METHODS

Primary amino acid sequence of WNT ligands and mutations

Sequences for all human WNT ligands were queried in UniProt (Database Uniprot: www.uniprot.org) and the *.clustal_num file imported into Jalview (v.2.11.3.2) (18) for further analysis. The COSMIC database (Database COSMIC: https://cancer.sanger.ac.uk/cosmic) (19) was interrogated in February and March 2024 to retrieve mutation data available for 18 WNT ligands with search term “WNT x” and tissue “large intestine.” We chose colorectal cancers (large intestine) since there is a direct correlation between mutations in WNT signaling and carcinogenesis (see above). The mutations identified in COSMIC were annotated on the aligned WNT amino acid sequence (see below).

Protein structure preparation

Protein structures were downloaded from the PDB or AlphaFold web server and prepared using FoldX v.5.0 (20). Structure files were minimized using the RepairPDB function in FoldX 5.0, applied 10 times, as performed previously (21). Minimizing the structure in this way results in convergence to an energy minima determined by the force field applied by FoldX through rearranging the amino acid side chains. As FoldX minimizes side chains by running through the sequence from C- to N-terminal amino acids, multiple repeats of the minimization allow it to reach a lower energy minima than just one due to the spatial organization of the protein (i.e., if residue 1 is near residue 100, then the second minimization will better optimize residue 1 to its neighbor 100 after it has also been optimized).

Structural energy calculations

Structural energy calculations (both $\Delta\Delta G$ and $\Delta\Delta G$ of binding) were performed using FoldX 5.0. We calculated the Gibbs free energy and Gibbs free energy of binding (ΔG and ΔG of binding); the Gibbs free energy is a measure of the thermal stability of the protein and protein complexes, respectively, being proportional to the entropic and enthalpic energy of the proteins. We compared the Gibbs free energy of the original wild-type (WT) protein/complex to a mutated protein being queried to determine the delta of the ΔG (denoted as $\Delta\Delta G$ and $\Delta\Delta G$ of binding). FoldX calculates predicted energies of mutations through the use of an empirical force field that includes energy terms for van der Waals interactions, electrostatic interactions, H-bonding, and entropic penalties that it uses to estimate the free energy of a structure. This yields positive or negative predicted free energy changes upon mutation that indicate a stabilizing or destabilizing effect. FoldX has been demonstrated to have a reasonable accuracy (R^2 against experimental $\Delta\Delta G$s between 0.29 and 0.73 depending on the quality of the protein structure) (22), and we have used it previously to gain structural insights into the effects of mutations on protein structures (14,15,21,23,24). Its computational efficiency—the ability to calculate $\Delta\Delta G$ of a single mutation in less than a minute—makes it more amenable to high-throughput calculations than simulation-based methods such as Rosetta’s cartesian_ddg (25). In each case, a saturation screen was performed by mutating every amino acid to every other amino acid and calculating the resultant change in the Gibbs free energy (ΔG). The pipeline and code for reproducing these methods have been published and demonstrated elsewhere (21,23), and all code for running these calculations is available in our GitHub repository (GitHub Data: https://github.com/shorthouse-lab/binding_ddg). For binding calculations, we mutated all residues in the WNT protein within 10 Å of the bound FZD receptor to every other amino acid. Residues within 10 Å were determined using Biopython (26). For binding $\Delta\Delta G$, we first used the BuildModel command in FoldX to generate a structure of the mutant form of the protein, followed by the AnalyseComplex command to calculate the change in energy. Scripts to reproduce these calculations are available on GitHub (GitHub Data: https://github.com/shorthouse-lab/binding_ddg), and data specifically for this manuscript are available in their own repository (GitHub Data: https://github.com/shorthouse-lab/WNTligand_stability).

TCGA data

The Cancer Genome Atlas (TCGA) is a collection of over 11,000 primary cancer samples that have been sequenced and characterized at a molecular level. These data are made available publicly, and we used data from the cohort of patients diagnosed with colorectal adenocarcinoma (27). Data

was downloaded from cBioPortal (Database cBioPortal:www.cbioportal.org) (28), selecting the survival data (overall survival), and mutation data for TCGA pan-cancer atlas colorectal (COADREAD) cohort.

dN/dS

The ratio of nonsynonymous to synonymous substitutions (dN/dS) analysis (29,30) was performed using the dndscv R package. dN/dS ratios are widely used as a measure of the evolutionary selection of genes. In our case, applying this to cancer, mutations with more synonymous versus nonsynonymous mutations than expected (a higher dN/dS ratio) are predicted to be under evolutionary selective pressure in the tumor cohort, and mutations occurring in these genes are likely to have functional consequences. The ratio was calculated by supplying the dnds function to the mutations observed in every patient in our TCGA cohort.

Survival analysis

Survival analysis was performed using the lifelines python package (31). We performed survival analysis based on overall survival (denoted "OS" in TCGA) and calculated the p value using the log rank test.

Selection calculation

Calculations of the expected and observed mutational properties were performed using Darwinian Shift (13), available at GitHub (GitHub Data: https://github.com/michaelhall28/darwinian_shift). Darwinian Shift estimates expected mutational rates by calculating the mutational signature of a set of cancer samples. This mutational signature is a summary of the proportion of different mutations occurring in given DNA contexts, which are linked to the mutational processes occurring in the tumor. Given the mutational signature, we calculate what amino acid mutations we would expect to see and overlay them on the structure, assuming an even distribution as would occur if no mutational selection is occurring, to calculate an "expected distribution" of mutations. We then compare this expected distribution to our observed distribution to determine an effect size and statistical significance. Statistical significance is determined through a Monte Carlo test with 100,000 permutations to determine whether the observed mutations are different from what we expect to observe, given an assumption of no evolutionary selection.

Elastic network modeling

Elastic network modeling was performed using the tool Prody (32) in python. In elastic network modeling, proteins are represented as a network of nodes (in this case, based on the α carbons), connected by harmonic springs to approximate the intrinsic flexibility of the structure. In this approach, interactions are defined between nodes within a specified cutoff distance (in this case, 1 nm) to establish the elastic network. The model then predicts the collective motions of the protein by computing the normal modes of the network. The resulting eigenvectors describe the predicted displacement patterns of the nodes relative to each other, capturing the large-scale conformational dynamics of the structure. The code used to generate the elastic network model is available in the GitHub repository associated with this manuscript and follows the tutorial laid out at the prody website (Prody Data: http://www.bahargroup.org/prody/tutorials/enm_analysis/gnm.html).

Molecular dynamics simulations

Molecular dynamics was performed using Gromacs v.2023 (33) and following the protocol available in (34). The protein structure was mutated using the FoldX BuildModel command and minimized by applying the RepairPDB command 10 times consecutively. The protein was further energy minimized using the optimized potentials for liquid simulations—all atoms (OPLS-AA) force field (35), which we chose to use because of its versatility. The systems were then set up by adding the protein to the center of a 10 × 10 × 10 nm cubic box, which ensures at least a 2 nm distance between periodic images of the protein, ensuring that periodic images are outside of the cutoff thresholds used for the simulations. Proteins were solvated with simple point charge (SPC) water molecules and counterions added, along with additional ions to make a molar concentration equal to 0.05 mol/L. The systems were then energy minimized again and equilibrated using constant number of particles, volume and temperature (NVT)and constant number of particles, pressure and temperature (NPT) ensembles and restrained α carbons. All stages used the .mdp files available at the MD Tutorials website (MdTutorials Data: http://www.mdtutorials.com/gmx/index.html) following the tutorial for simulating lysozyme. The systems were then run for 200 ns of unrestrained equilibrium molecular dynamics. Analysis was performed by calculating the root mean-square deviation, root mean-square deviation, and principal components using the python library mdtraj (36). We followed the protocol outlined by Farmer et al. (37) for plotting statistical significance of the differences per residue.

Code to repeat the analysis in this manuscript is available at a GitHub repository for this publication(GitHub Data: https://github.com/shorthouse-lab/WNTligand_stability).

RESULTS AND DISCUSSION

Mutations to WNT ligands in cancer show tissue and protein-sequence specificity

We first performed a pan-cancer analysis of mutations to WNT ligand genes in the COSMIC database (Fig. 1). WNT genes were tested for mutations in ~45,000 samples across the COSMIC database (45,245 ± 158, median samples per WNT ± SD). WNT7A showed the highest (1.7%) and WNT1 the lowest (0.38%) frequency of mutations across all cancers listed on COSMIC. Given the established importance of WNT signaling in colorectal cancers, we subset COSMIC into samples taken from cancers of the large and small intestines. Our analysis uncovers a significant tissue specificity of mutations to WNT ligands, as occurrences are 18.5-fold less frequent in cancers of the small intestine compared to the large intestine (median ± SD of 2 ± 1 vs. 37 ± 15 for small and large intestine mutations, respectively, $p < 0.0001$, Mann-Whitney test; Fig. S1).

For samples from cancer of the large intestine (colorectal cancer), 0.79% contained mutations in a WNT gene; for comparison, KRAS is mutated in 32% (in 80,932 unique samples). The C>T mutation, considered to be driven by endogenous mutational processes, was found to be most prevalent, whereas the C>A mutation was less prevalent in the WNT ligand genes (Fig. S2).

We next performed a sequence alignment of all 19 human WNT proteins. Numerous conserved amino acid residues can be identified forming conserved motifs (Fig. 1). We identify a number of highly conserved cysteine residue motifs that are repeated 22 times throughout the primary sequence of all WNT proteins, which are known to be essential for maintaining WNT structure. Seven cationic amino acid (arginine and/or lysine) motifs, of which 2

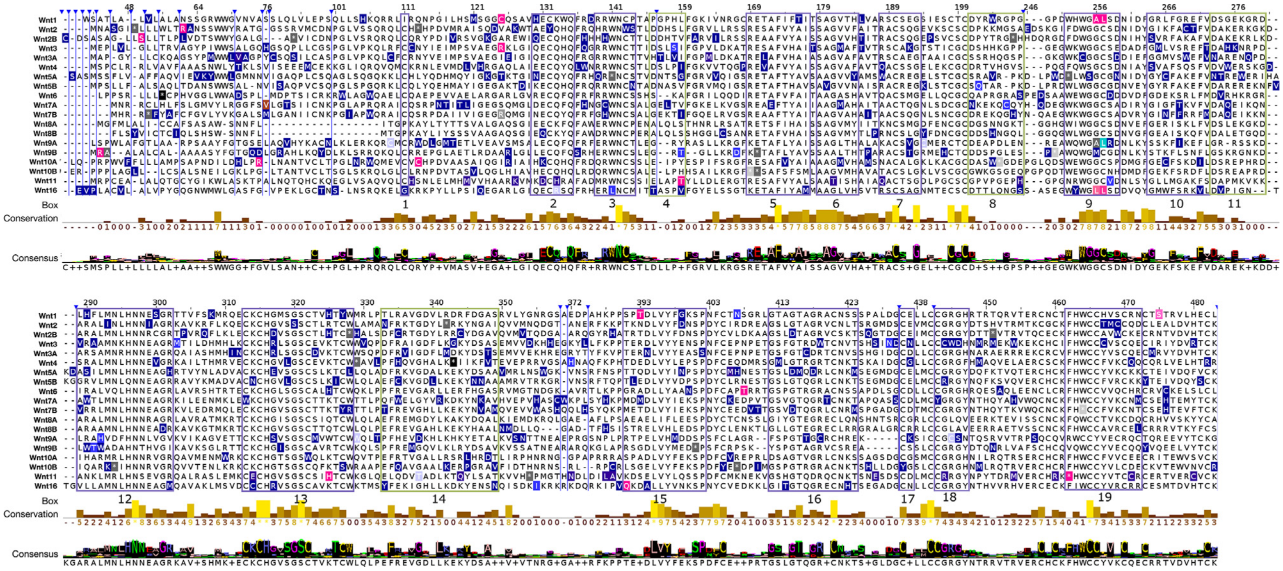

FIGURE 1 Sequence alignment of human WNT proteins. Conserved motifs are highlighted and numbered (*boxes 1–19*). Locations of missense mutations are highlighted with colors to indicate mutations found in COSMIC for cancer of the large intestine (*dark blue*), small intestine (*light blue*), or both (*pink*). Vertical blue lines with arrowheads indicate truncated Wnt sequences shown here for clarity.

arginine residues are at positions 140 and 418, are present in all WNT sequences; eight anionic amino acids (glutamate and/or aspartate) are conserved across all WNT genes. There are also a number of neutral amino acids that show high conservation, for example, glycine (at positions 193, 301, 317, 320, 415, and 444), phenylalanine (at positions 173 and 270), and tyrosine (at positions 141, 254, and 328). In addition, there are at least three serine repeats (at positions 319, 321, and 401), as well as highly conserved alanine, asparagine, leucine, histidine, proline, and valine residues (Fig. 1).

The conserved, individual amino acid sequences and motifs (e.g., RWNC, CKCHG, SGSC, R/K/Q TCW, and H/I/Q,R WCC) in the WNT proteins are shown in purple boxes 1–19 in Fig. 1. Of particular interest are box 13, which contains the highly conserved and mutated SGSC thumb domain motif, and box 19, which contains the relatively sparsely mutated and highly conserved FHWCC*V motif of the finger domain (38). A structure of a WNT ligand bound to a FZD cysteine-rich region is shown in Fig. S3. We next used these identified motifs to target an exploration of the predicted functional consequences mutations to WNT ligands in colorectal cancers.

Mutations in colorectal cancers do not have significant functional effects on WNT/FZD binding

Given that WNT binding is a key event in the signaling cascade and that this cascade is activated in colorectal cancer, we surmised that mutations may alter the binding energy of the WNT ligand for its FZD receptor. Supporting this hypothesis, we identified a number of mutations in the regions of the ligand responsible for binding (Fig. 2 *A*). We calculated the solvent accessible surface area (SASA) of each residue involved in the thumb and finger domains (box 13 and box 19) and find that there is no obvious correlation between the SASA and mutation rate. This is likely related to the structure of the domains, which are extended and contain exposed β sheets, meaning there are no "buried" residues (SASA $\leq$ 20 Å^2). We next performed an in silico saturation mutagenesis screen for the energy change ($\Delta\Delta G$) of binding for every possible mutation in the so-called thumb (*box 13* of Fig. 1) and index finger (*box 19* of Fig. 1) domains of the WNT/FZD interaction from the crystal structure of human WNT3 bound to murine FZD8 (PDB: 6AHY) (11,38). While the structure studied is that of murine FZD8, there is a 100% sequence identity to the human gene in the region covered (Fig. S4). Saturation mutagenesis reveals that a remarkably low number of residues are actually responsible for maintaining the binding of the WNT/FZD complex, in strong agreement with experimental work from Kumar et al. (17), who mutated some of these residues to alanine to study the effects on binding. We also validate these findings by performing a 200 ns molecular dynamics simulation of the WNT3/FZD8 complex, determining the residence time of interactions (defined as percentage time within 4 Å) between studied residues (Fig. S5). Within the highly conserved SGCG motif, palmitoylation of the serine residue allows the attached fatty chain to insert into a groove in FZD to facilitate binding and increases membrane residence (11,38). $\Delta\Delta G$ calculations demonstrate that only mutations to the glycine and cysteine residues in this motif are predicted to consistently impact binding energies (Fig. 2 *B*). We calculated the

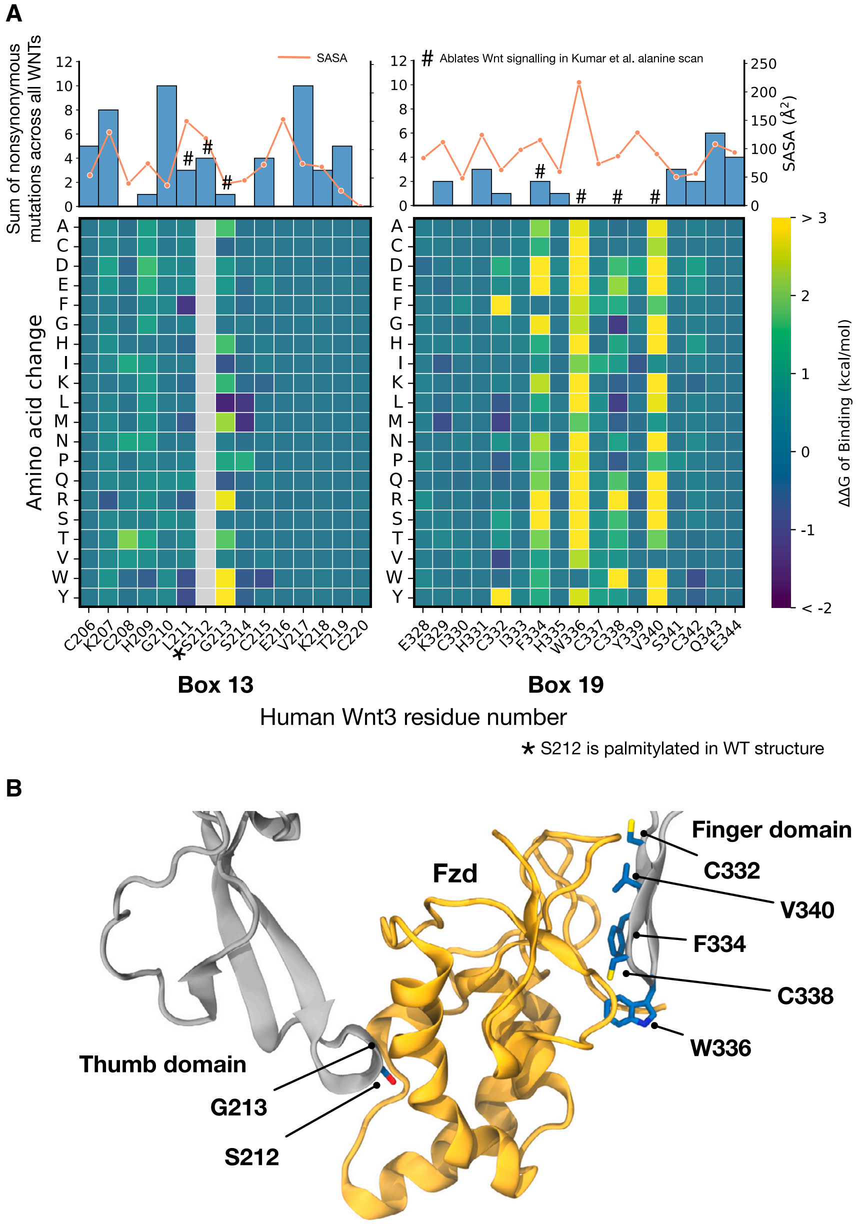

FIGURE 2 In silico mutagenesis screen of mutations to WNT-FZD binding. (*A*) Saturation mutagenesis screen result for binding energy (ΔΔG of binding) calculated for the crystal structure of human WNT3a bound to murine FZD8 (PDB: 6AHY) showing (*top*) the sum of nonsynonymous mutations by aligned sequence position across all WNTs in COSMIC. Blue bars represent the number of mutations, and the orange line represents the solvent accessible surface area (SASA). (*Bottom*) ΔΔG of binding per mutation in each interacting residue. (*Left*) Thumb domain (*box 13* in Fig. 1) and (*right*) finger domain (*box 19* in Fig. 1). (*B*) Interaction of WNT-FZD highlighting major residues involved in binding. Numbering refers to human WNT3a.

number of mutations in our cohort to these residues, counting any missense mutation to the position in the sequence alignment for any WNT. Mutations to these residues are rare in our cohort, suggesting that through the survivorship bias (30) or other processes, mutations to this region that impact binding are not enriched in colorectal cancers. Similarly, for the finger domain, predicted binding energies show large changes upon mutation to components of the FHWCC*V conserved sequence, particularly the phenylalanine, tryptophan, and valine. Again, we find that residues contributing to binding interactions are devoid of mutations, suggesting that mutations to these regions of the structure are not enriched in colorectal cancer, including mutations that are expected to increase binding affinity. We additionally performed a multiple sequence alignment of all 10 human FZD sequences and studied the conservation of the interaction site with the WNT thumb and finger domains (Fig. S6). The thumb domain binds a conserved sequence motif, and the finger domain binds to a series of conserved cysteines, as has been identified previously (9), supporting the hypothesis that the cysteines within the cysteine loops of FZD are the “core” binding partners. Having determined that mutations are generally not occurring in the residues responsible for WNTs binding their FZD coreceptor, we looked to other structural features to identify if and how mutations in colorectal cancers may be impacting WNTs.

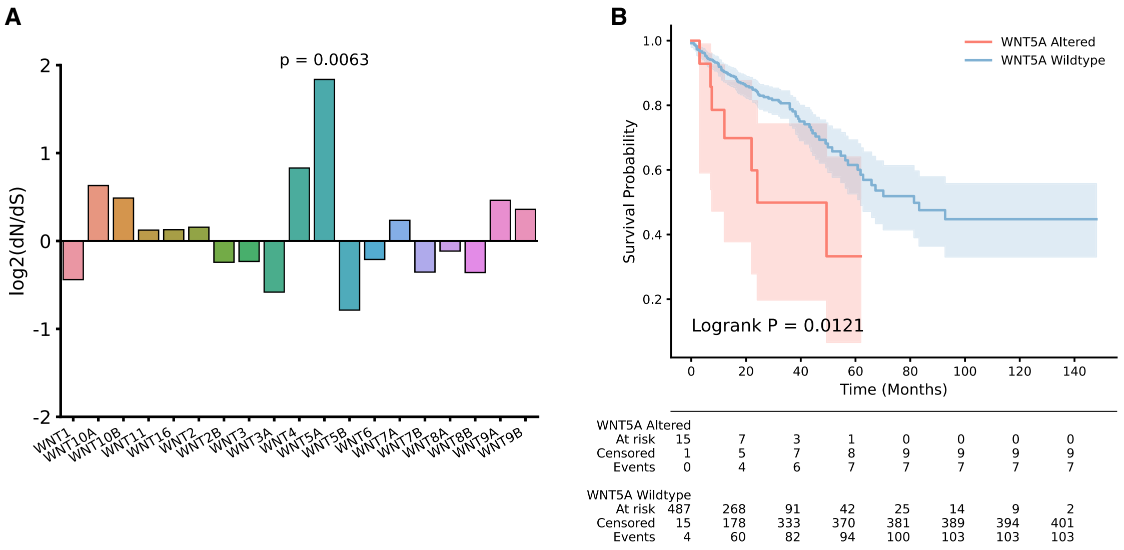

FIGURE 3 WNTs contribute to tumor phenotype in colorectal adenocarcinoma. (*A*) Log2 dN/dS ratios for all 19 WNTs calculated for 592 patients in TCGA colorectal cancer data set. *p* value represents Maximum-Likelihood test. (*B*) Kaplan-Meier analysis of 502 patients from TCGA split into patients WT for WNT5A (blue) and genetically altered (red). *p* value represents Logrank test.

Mutations in WNTs in human colorectal cancer are under evolutionary selection

We next attempted to identify WNT ligands that are under mutational selection in human colorectal cancers. We first performed dN/dS analysis (10) on mutation data for 592 patients with colorectal adenocarcinoma in TCGA pan-cancer atlas (27). dN/dS compares ratios of synonymous and nonsynonymous mutations, corrects for the mutational signature the samples are exposed to, and generates a corrected ratio that determines how much each gene is under selection in the cohort (Fig. 3 *A*). When honing specifically on WNT genes, many exhibit log2(dN/dS) ratios different from 0 (0 is expected if the genes are not under evolutionary selection). WNT5A, in particular, demonstrates a significant ($p = 0.0063$) log2(dN/dS) ratio of 1.84, suggesting it is under positive selection (i.e., more mutated than expected by chance).

Kaplan-Meier analysis on patients for whom both mutational and survival data were available ($n = 502$) shows that patients with mutations in WNT5A ($n = 15$, defined as those with a nonsynonymous mutation or copy-number alteration) had a significantly worse prognosis than WT equivalent patients (log rank $p = 0.0121$) (Fig. 3 *B*). That these mutations are under selection and correlate with outcome suggests that they may have functional consequences on the structure.

Mutations in WNT5A in human colorectal cancer alter protein structural dynamics

Having identified that WNT5A is under selection and that mutations correlate with patient outcome in colorectal cancers, we next sought to understand how they might impact the activity of the protein. We extracted all mutations in WNT5A from samples of colorectal cancer from the COSMIC database (which is a large pool of mutations, including the subset previously analyzed in TCGA). Mutations occur throughout the primary sequence of the protein (Fig. 4 *A*); the most common single mutation is a change of valine at position 310 to methionine (V310M), which, to our knowledge, has not yet been characterized. Applying the nonrandom mutational clustering algorithm (39) to identify statistically significant one-dimensional clusters of mutations in the primary sequence, we identify two regions of statistically unlikely mutational clusters—one covering residues 182–184 and one covering residues 310–322. Given that there are characterized structures of human WNTs, we used the AlphaFold (12)-predicted structure of human WNT5A for further analysis.

The WNT5A structure was analyzed using a pipeline for studying mutational selection, which involved energy minimizing the structure before calculating the properties of mutations to study selection. We performed a computational saturation mutagenesis screen of the whole protein to calculate mutational energies and found that according to statistics calculated through the Darwinian Shift method, mutations observed in colorectal cancers are not higher or lower energy than expected by chance (Monte Carlo $p = 0.491$; Fig. S7).

Having surmised that mutations in WNT5A in colorectal cancers do not significantly impact protein stability/folding energy, we sought to study how they may impact the dynamics of the protein. As the WNT structure contains two extended domains important for binding the partner FZD receptor family, we hypothesized that mutations may impact

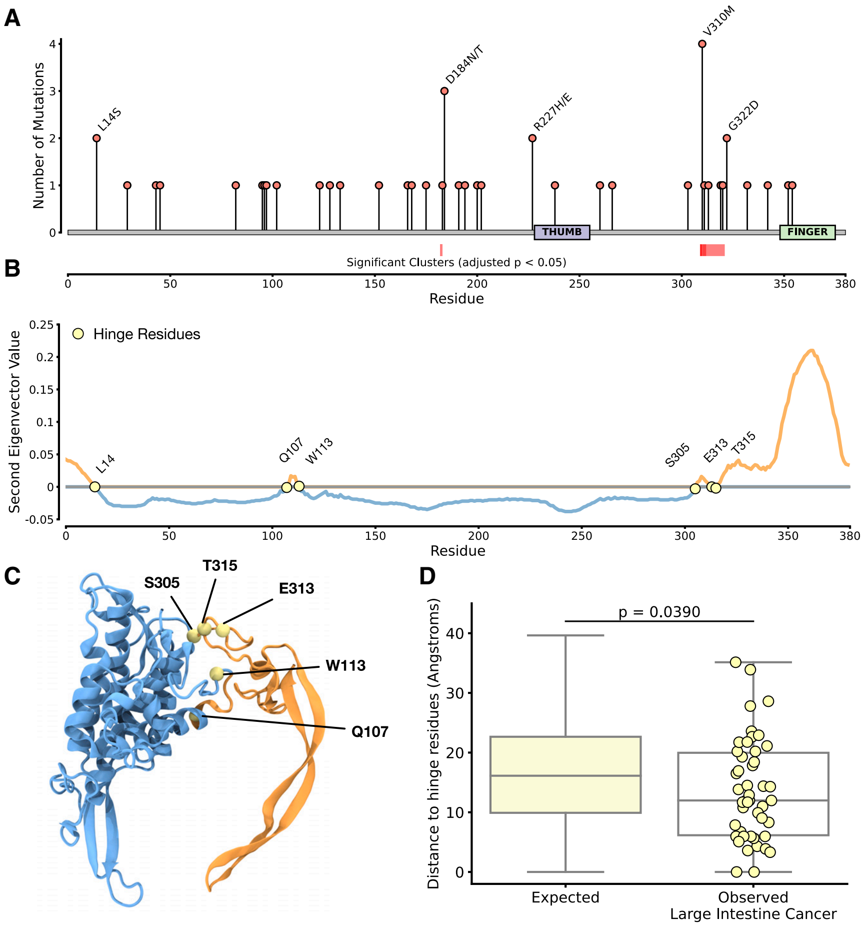

FIGURE 4 WNT5A mutations from colorectal cancers are statistically close to identified hinge regions. (*A*) Lollipop plot of WNT5A mutations occurring in human colorectal cancers; the mutations are extracted from COSMIC. Red bars beneath the *x* axis represent regions of statistically significant mutational clustering as determined by nonrandom mutational clustering (NMC). (*B*) Second eigenvector value of protein dynamics as calculated by prody. Highlighted residues represent identified hinge points (regions where the eigenvector is 0). (*C*) Structure of WNT5A colored by eigenvector (*blue*: positive, *orange*: negative), with hinge residues highlighted. (*D*) Comparison of distance of observed mutations in colorectal cancers from hinge residues to an expected distribution based on the mutational signature. *p* represents the Monte Carlo test performed by Darwinian Shift.

the dynamics of these domains. We performed elastic network modeling of the protein using prody (32) (Fig. 4, *B* and *C*), which calculates the predicted global motions of the structure based on elastic links formed between α carbons of the protein. The first eigenvector identified corresponded to the movement of the unstructured first 40 amino acids of the structure; however, the second eigenvector uncovered a "hinging" motion representing the dynamics of the finger and thumb domains. Extracting predicted hinge points from this analysis (amino acids for which the eigenvalues are 0) identified residues L14, Q107, W113, S305, E313, and T315. Notably, these residues overlap with the identified cluster of mutations at residues 310–322.

Calculating expected distances from these hinge residues, the observed mutations are statistically significantly (Monte Carlo $p = 0.039$) closer to these hinge residues than expected (Fig. 4 *D*) and, therefore, are expected to functionally impact the dynamics of the finger and thumb domains of WNT5A.

To validate these results, we next performed 200 ns equilibrium atomistic molecular dynamics simulations of the WT and the most common (V310M) mutation to the hinge domain. Simulations quickly reach equilibrium during unrestrained molecular dynamics. Calculating the per-residue root mean-square fluctuation, as well as the root mean-square deviation of the final 150 ns (i.e., those occurring after the initial relaxation of the structure) (Figs. 5 and S8), along with principal components of the whole trajectory, shows that there is increased structural variability and motility of the mutant WNT compared to the WT, despite them both eventually reaching a similar energy minima (Fig. S8). This demonstrates that mutation V310M, the most common hinge mutation observed in human colorectal cancer, is likely to increase the flexibility and conformational space accessible to WNT ligands, and this may allow it increased ability to bind to its partner FZD receptors.

CONCLUSION

This is an initial, detailed analysis of the putative structure-function relationship of WNT ligands using a toolkit previously developed in our laboratory to determine the functionality of mutations in a protein (13,21). This is also a detailed amalgamation of mutations found in WNT proteins in colorectal cancer.

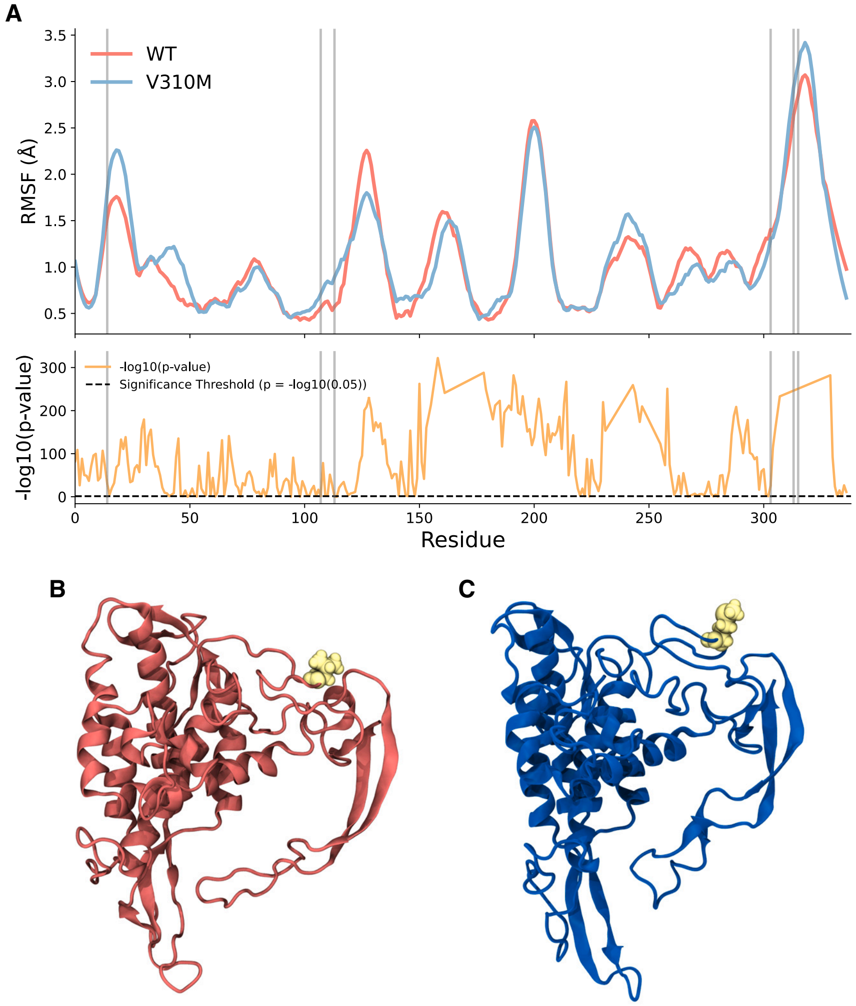

FIGURE 5 (*A*) Per-residue root mean-square fluctuation (RMSF) calculations for the final 150 ns of equilibrium molecular dynamics simulations of wild-type (*red*) and V310M mutant (*blue*) human WNT5A. (*Top*) RMSF per residue in the structure. (*Bottom*) $-\log 10(p$ value) for the difference in the RMSFs. p value represents a Two-sample t test. Gray vertical lines represent amino acids identified as hinge sites by prody. (*B* and *C*) Structures of WT WNT5A (*red*) and V310M mutant (*blue*) from the trajectories.

Assessing the functional effect of individual mutations using conventional in vitro approaches is a monumental task. Our approach of performing saturation mutagenesis screens and comparing sets of mutations against expected distributions provides a convenient, medium-throughput in silico analytical tool. While this method is less accurate than, for example, dynamics-based "alchemical" calculations (40), it provides a useful approach for computationally feasible saturation screens, which can be used as the basis for statistical and further exploratory analysis. After identifying all the mutations in the 18 WNT ligands in colorectal cancer, we have used two paradigm examples to demonstrate the feasibility of this approach to assess the putative functional manifestation of structural mutations in WNT ligands. In our first example, we use binding energy calculations to uncover that mutations in our data set do not occur in the regions of the protein responsible for binding FZD, and in our second example, we focus on a single WNT (human WNT5A) to show that mutations in colorectal cancer are expected to alter the ligand conformational flexibility.

In a number of colorectal cancers, a mutation in the APC gene appears to be a key carcinogenic factor (1). Mutations in the APC gene have been cataloged in nearly 50% of colorectal cancers, while mutations in WNT ligand genes are present in nearly 14% of colorectal cancers. We find preliminary evidence that the impact of mutations in WNT ligands could be similar to APC mutations. Mutations are predicted to increase the likelihood of binding of WNT ligands to their putative receptors, resulting in prolonged or constitutive activation of WNT signaling. We demonstrate that, perhaps counterintuitively, WNT ligands do not appear to be under evolutionary selection in colorectal cancer for mutations that alter binding energy to FZD receptors. Instead, we find evidence supporting mutational selection for variants that change the flexibility and dynamics of the protein and may impact the kinetics of it binding the receptor. This is similar to previous analysis performed by us (23), which demonstrated that pathogenic mutations in the enzyme fumarate hydratase exhibit a range of effects on the protein, one of which is to alter the dynamics of hinges within the structure.

We believe that this investigation provides a starting point for future work that will explore the impact and phenotypic roles of WNT ligand mutations in human cancers. While this work is computational, previous work using these techniques performed by us and others demonstrates that these workflows provide useful biological and functional insights into mechanisms of mutational impact on proteins.

AUTHOR CONTRIBUTIONS

Conceptualization, D.S. and A.A.; methodology, D.S. and A.A.; software, D.S. and A.A.; investigation, D.S. and A.A.; data curation, D.S. and A.A.; writing – original draft, D.S. and A.A.; writing – review & editing, D.S. and A.A.

DECLARATION OF INTERESTS

The authors declare no competing interests.

SUPPORTING MATERIAL

Supporting material can be found online at https://doi.org/10.1016/j.bpj.2025.03.030.

REFERENCES

1. Polakis, P. 2012. WNT Signaling in Cancer. *Cold Spring Harbor Perspect. Biol.* 4:a008052.
2. Ashmore, J., H. Olsen, …, A. Ahmed. 2019. WNTs control membrane potential in mammalian cancer cells. *J. Physiol. (Paris).* 597:5899–5914. https://doi.org/10.1113/JP278661.
3. Kühl, M., L. C. Sheldahl, …, R. T. Moon. 2000. The WNT/Ca2+ pathway A new vertebrate WNT signaling pathway takes shape. *Trends Genet.* 16:279–283. https://doi.org/10.1016/S0168-9525(00)02028-X.
4. Nusse, R., and H. Clevers. 2017. WNT/β-Catenin Signaling, Disease, and Emerging Therapeutic Modalities. *Cell.* 169:985–999. https://doi.org/10.1016/j.cell.2017.05.016.
5. Thrasivoulou, C., M. Millar, and A. Ahmed. 2013. Activation of intracellular calcium by multiple WNT ligands and translocation of β-catenin into the nucleus: A convergent model of WNT/Ca2+and WNT/β-catenin pathways. *J. Biol. Chem.* 288:35651–35659. https://doi.org/10.1074/jbc.M112.437913.
6. Croce, J. C., and D. R. McClay. 2008. Evolution of the WNT pathways. *Methods Mol. Biol.* 469:3–18. https://doi.org/10.1007/978-1-60327-469-2_1.
7. Martin-Orozco, E., A. Sanchez-Fernandez, …, M. Ayala-San Nicolas. 2019. WNT Signaling in Tumors: The Way to Evade Drugs and Immunity. *Front. Immunol.* 10:2854. https://doi.org/10.3389/fimmu.2019.02854.
8. Zhan, T., N. Rindtorff, and M. Boutros. 2017. WNT signaling in cancer. *Oncogene.* 36:1461–1473. https://doi.org/10.1038/onc.2016.304.
9. Agostino, M., S. Ö. G. Pohl, and A. Dharmarajan. 2017. Structure-based prediction of WNT binding affinities for frizzled-type cysteine-rich domains. *J. Biol. Chem.* 292:11218–11229. https://doi.org/10.1074/jbc.M117.786269.
10. Yang, S., Y. Wu, …, F. Xu. 2018. Crystal structure of the Frizzled 4 receptor in a ligand-free state. *Nature.* 560:666–670. https://doi.org/10.1038/s41586-018-0447-x.
11. Hirai, H., K. Matoba, …, J. Takagi. 2019. Crystal structure of a mammalian WNT–frizzled complex. *Nat. Struct. Mol. Biol.* 26:372–379. https://doi.org/10.1038/s41594-019-0216-z.
12. Jumper, J., R. Evans, …, D. Hassabis. 2021. Highly accurate protein structure prediction with AlphaFold. *Nature.* 596:583–589. https://doi.org/10.1038/s41586-021-03819-2.
13. Hall, M. W. J., D. Shorthouse, …, B. A. Hall. 2023. Mutations observed in somatic evolution reveal underlying gene mechanisms. *Commun. Biol.* 6:753.
14. Shorthouse, D., and B. A. Hall. 2021. SARS-CoV-2 Variants Are Selecting for Spike Protein Mutations That Increase Protein Stability. *J. Chem. Inf. Model.* 61:4152–4155. https://doi.org/10.1021/acs.jcim.1c00990.
15. Fowler, J. C., C. King, …, P. H. Jones. 2021. Selection of Oncogenic Mutant Clones in Normal Human Skin Varies with Body Site. *Cancer Discov.* 11:340–361.
16. Shorthouse, D., H. Lister, …, B. A. Hall. 2024. Understanding large scale sequencing datasets through changes to protein folding. *Brief. Funct. Genomics.* 23:517–524. https://doi.org/10.1093/bfgp/elae007.
17. Kumar, S., M. Žigman, …, S. Özbek. 2014. Molecular dissection of WNT3a-Frizzled8 interaction reveals essential and modulatory determinants of WNT signaling activity. *BMC Biol.* 12:44. https://doi.org/10.1186/1741-7007-12-44.
18. Waterhouse, A. M., J. B. Procter, …, G. J. Barton. 2009. Jalview Version 2-A multiple sequence alignment editor and analysis workbench. *Bioinformatics.* 25:1189–1191. https://doi.org/10.1093/bioinformatics/btp033.
19. Tate, J. G., S. Bamford, …, S. A. Forbes. 2019. COSMIC: The Catalogue Of Somatic Mutations In Cancer. *Nucleic Acids Res.* 47:D941–D947. https://doi.org/10.1093/nar/gky1015.
20. Delgado, J., L. G. Radusky, …, L. Serrano. 2019. FoldX 5.0: working with RNA, small molecules and a new graphical interface. *Bioinformatics.* 35:4168–4169.
21. Jamshidi Parvar, S., B. A. Hall, and D. Shorthouse. 2024. Interpreting the effect of mutations to protein binding sites from large-scale genomic screens. *Methods.* 222:122–132. https://doi.org/10.1016/j.ymeth.2023.12.008.
22. Buß, O., J. Rudat, and K. Ochsenreither. 2018. FoldX as Protein Engineering Tool: Better Than Random Based Approaches? *Comput. Struct. Biotechnol. J.* 16:25–33.
23. Shorthouse, D., M. W. J. Hall, and B. A. Hall. 2021. Computational Saturation Screen Reveals the Landscape of Mutations in Human Fumarate Hydratase. *J. Chem. Inf. Model.* 61:1970–1980.
24. Lee, M., D. Shorthouse, …, A. R. Venkitaraman. 2021. Cancer-causing *BRCA2* missense mutations disrupt an intracellular protein assembly mechanism to disable genome maintenance. *Nucleic Acids Res.* 49:5588–5604.
25. Kellogg, E. H., A. Leaver-Fay, and D. Baker. 2011. Role of conformational sampling in computing mutation-induced changes in protein structure and stability. *Proteins.* 79:830–838.
26. Cock, P. J. A., T. Antao, …, M. J. L. de Hoon. 2009. Biopython: freely available Python tools for computational molecular biology and bioinformatics. *Bioinformatics.* 25:1422–1423.
27. Weinstein, J. N., E. A. Collisson, …, T. Kling. 2013. The cancer genome atlas pan-cancer analysis project. *Nat. Genet.* 45:1113–1120. https://doi.org/10.1038/ng.2764.
28. Cerami, E., J. Gao, …, N. Schultz. 2012. The cBio Cancer Genomics Portal: An open platform for exploring multidimensional cancer genomics data. *Cancer Discov.* 2:401–404. https://doi.org/10.1158/2159-8290.CD-12-0095.
29. Martincorena, I., K. M. Raine, …, P. J. Campbell. 2017. Universal Patterns of Selection in Cancer and Somatic Tissues. *Cell.* 171:1029–1041.e21.
30. Yang, Z., and J. Bielawski. 2000. Statistical methods for detecting molecular adaptation. *Trends Ecol. Evol.* 15:496–503. https://doi.org/10.1016/S0169-5347(00)01994-7.
31. Davidson-Pilon, C. 2019. lifelines: survival analysis in Python. *J. Open Source Softw.* 4:1317. https://doi.org/10.21105/joss.01317.

32. Bakan, A., L. M. Meireles, and I. Bahar. 2011. ProDy: Protein Dynamics Inferred from Theory and Experiments. *Bioinformatics.* 27:1575–1577.

33. Abraham, M. J., T. Murtola, …, E. Lindahl. 2015. GROMACS: High performance molecular simulations through multi-level parallelism from laptops to supercomputers. *SoftwareX.* 1–2:19–25.

34. Lemkul, J. 2019. From Proteins to Perturbed Hamiltonians: A Suite of Tutorials for the GROMACS-2018 Molecular Simulation Package [Article v1.0]. *Living J. Comput. Mol. Sci.* 1:5068. https://doi.org/10.33011/livecoms.1.1.5068.

35. Robertson, M. J., J. Tirado-Rives, and W. L. Jorgensen. 2015. Improved Peptide and Protein Torsional Energetics with the OPLS-AA Force Field. *J. Chem. Theor. Comput.* 11:3499–3509. https://doi.org/10.1021/acs.jctc.5b00356.

36. McGibbon, R. T., K. A. Beauchamp, …, V. S. Pande. 2015. MDTraj: A Modern Open Library for the Analysis of Molecular Dynamics Trajectories. *Biophys. J.* 109:1528–1532. https://doi.org/10.1016/j.bpj.2015.08.015.

37. Farmer, J., F. Kanwal, …, D. J. Jacobs. 2017. Statistical measures to quantify similarity between molecular dynamics simulation trajectories. *Entropy.* 19:646. https://doi.org/10.3390/e19120646.

38. Janda, C. Y., D. Waghray, …, K. C. Garcia. 2012. Structural basis of WNT recognition by frizzled. *Science.* 337:59–64. https://doi.org/10.1126/science.1222879.

39. Ye, J., A. Pavlicek, …, C.-H. Teng. 2010. Statistical method on nonrandom clustering with application to somatic mutations in cancer. *BMC Bioinf.* 11:11.

40. Seeliger, D., and B. L. de Groot. 2010. Protein thermostability calculations using alchemical free energy simulations. *Biophys. J.* 98:2309–2316. https://doi.org/10.1016/j.bpj.2010.01.051.

Biophysical Journal, Volume *124*

Supplemental information

Predicted functional consequences of WNT ligand mutations in color-ectal cancer

Aamir Ahmed and David Shorthouse

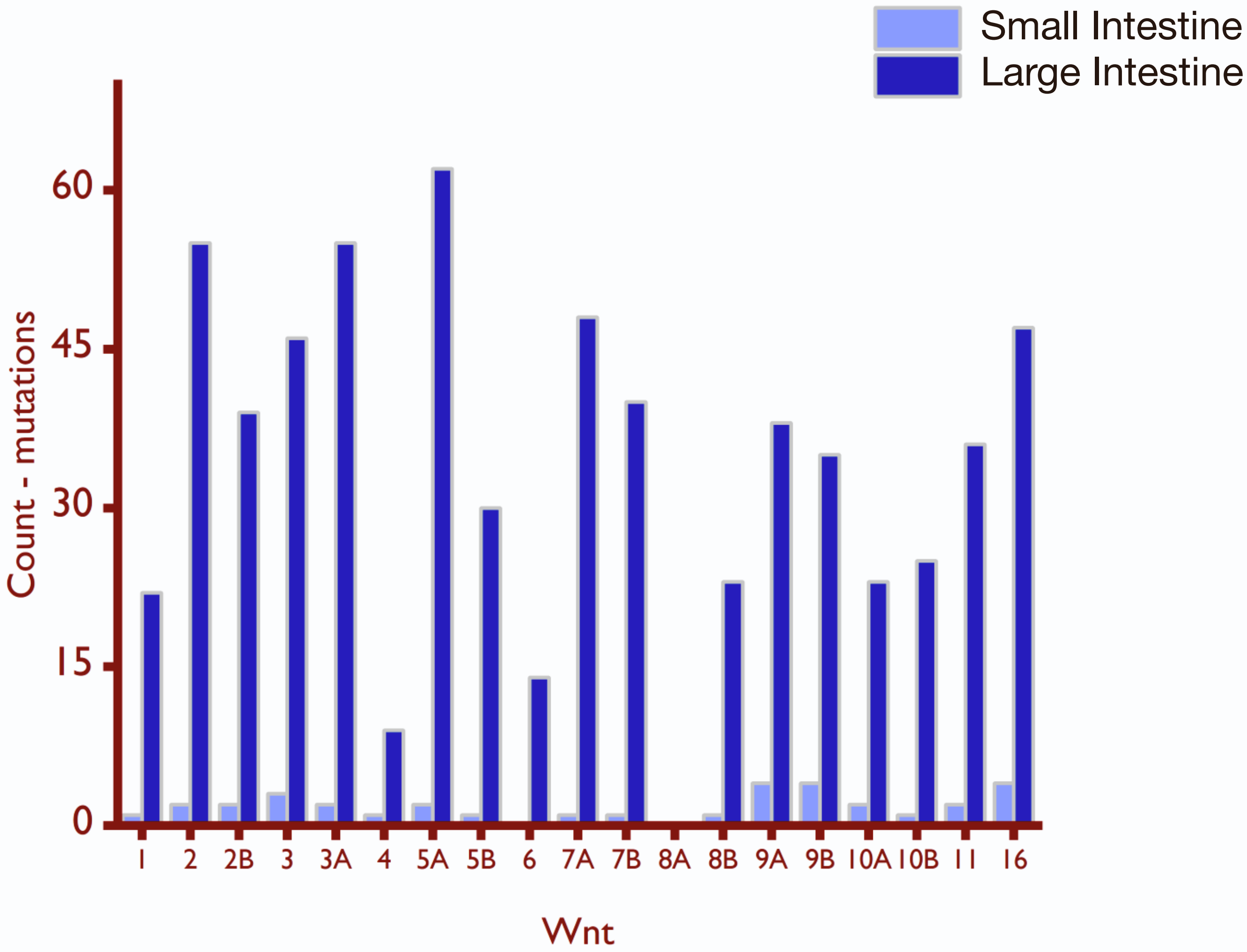

Figure S1: Mutational frequency of human Wnt genes in human cancers of the Large Intestine (dark blue), and Small Intestine (light blue). Data taken from COSMIC.

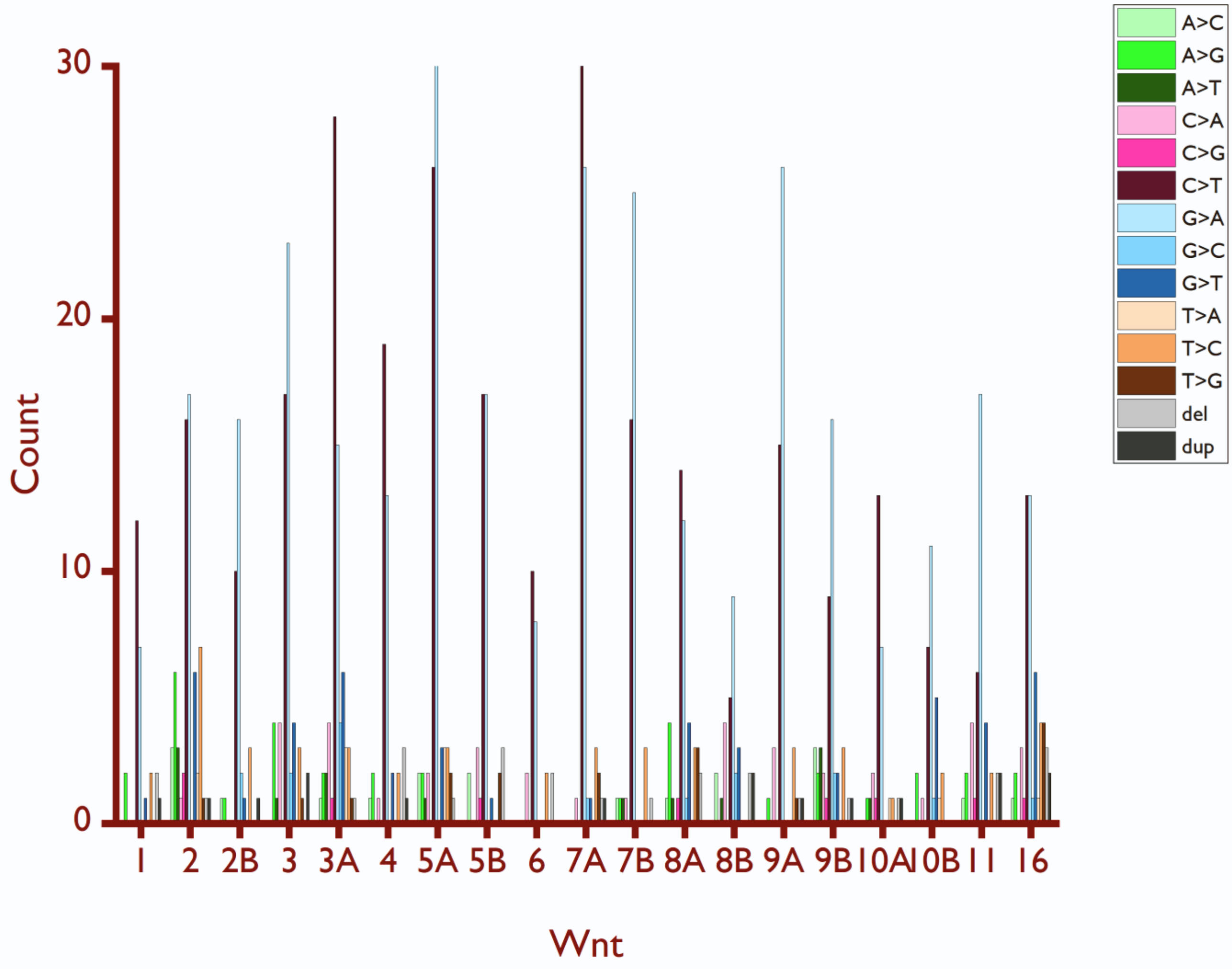

Figure S2: Mutational signatures found in Wnt genes in cancers of the Large Intestine. Data taken from COSMIC.

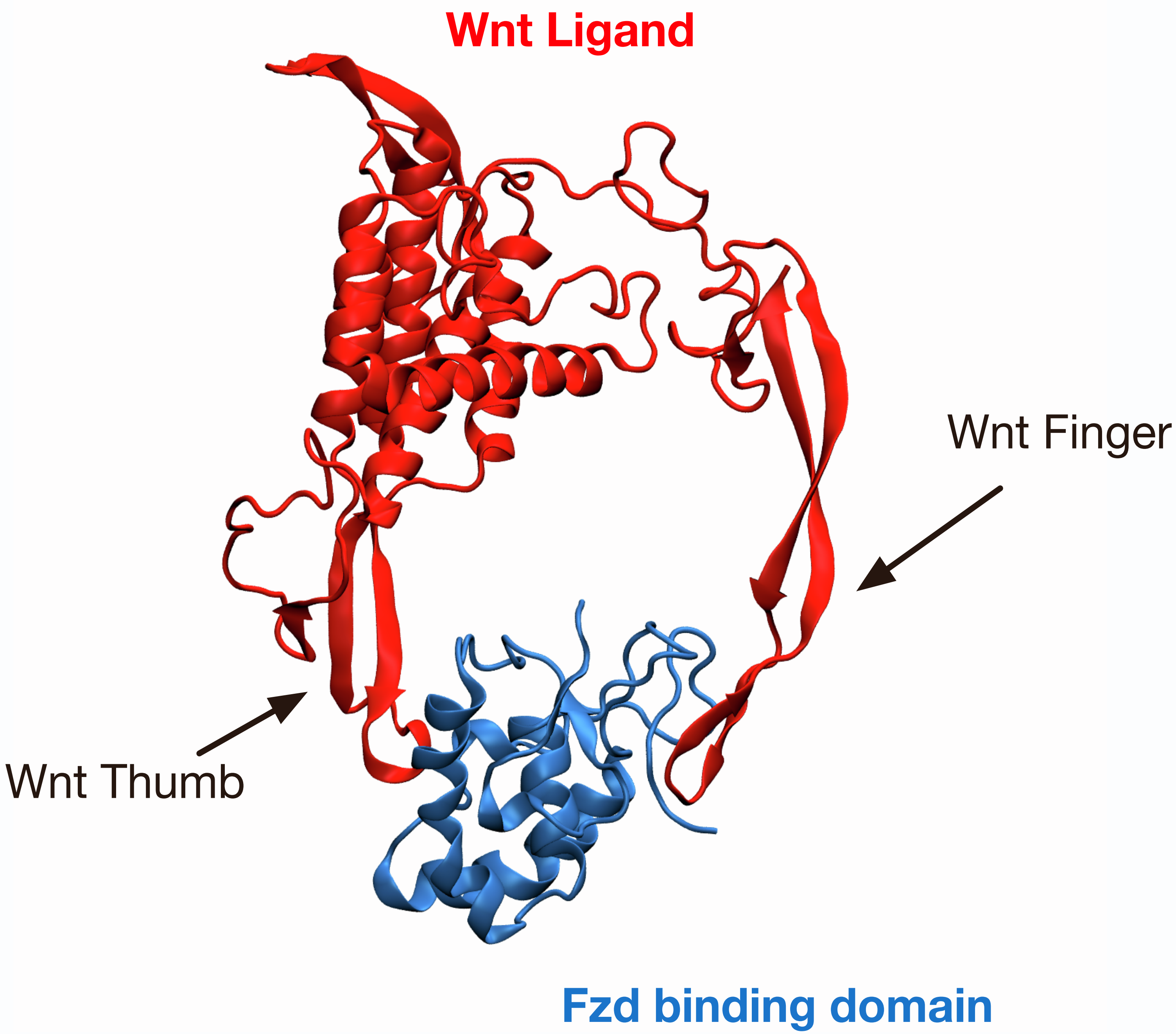

Figure S3: Structure of the Wnt/Fzd interaction, highlighting the Thumb and Finger domains.

```
Length: 696
Identity:     664/696 (95.4%)
Similarity:   667/696 (95.8%)
Gaps:          13/696 ( 1.9%)
```

- Covered in Crystal Structure 6AHY

```
sp|Q9H461|FZD8_HUMAN      MEWGYLLEVTSLLAALALLQRSSGAAAASAKELACQEITVPLCKGIGYNYTYMPNQFNHD   60
sp|Q61091|FZD8_MOUSE      MEWGYLLEVTSLLAALAVLQRSSGAAAASAKELACQEITVPLCKGIGYNYTYMPNQFNHD   60
                          *****************:******************************************

sp|Q9H461|FZD8_HUMAN      TQDEAGLEVHQFWPLVEIQCSPDLKFFLCSMYTPICLEDYKKPLPPCRSVCERAKAGCAP   120
sp|Q61091|FZD8_MOUSE      TQDEAGLEVHQFWPLVEIQCSPDLKFFLCSMYTPICLEDYKKPLPPCRSVCERAKAGCAP   120
                          ************************************************************

sp|Q9H461|FZD8_HUMAN      LMRQYGFAWPDRMRCDRLPEQGNPDTLCMDYNRTDLTTAAPSPPRRLPP-PPPGEQPPSG   179
sp|Q61091|FZD8_MOUSE      LMRQYGFAWPDRMRCDRLPEQGNPDTLCMDYNRTDLTTAAPSPPRRLPPPPPPGEQPPSG   180
                          ************************************************* **********

sp|Q9H461|FZD8_HUMAN      SGHGRPPGARPPHRGGGRGGGGGDAAAPPARGGGGGGKARPPGGGAAPCEPGCQCRAPMV   239
sp|Q61091|FZD8_MOUSE      SGHSRPPGARPPHRGGSSRGSGDAAAAPPSRG----GKARPPGGGAAPCEPGCQCRAPMV  236
                          ***.************.  *.*. *****:**    ************************

sp|Q9H461|FZD8_HUMAN      SVSSERHPLYNRVKTGQIANCALPCHNPFFSQDERAFTVFWIGLWSVLCFVSTFATVSTF   299
sp|Q61091|FZD8_MOUSE      SVSSERHPLYNRVKTGQIANCALPCHNPFFSQDERAFTVFWIGLWSVLCFVSTFATVSTF   296
                          ************************************************************

sp|Q9H461|FZD8_HUMAN      LIDMERFKYPERPIIFLSACYLFVSVGYLVRLVAGHEKVACSGGAPGAGGAGGAGGA-AA   358
sp|Q61091|FZD8_MOUSE      LIDMERFKYPERPIIFLSACYLFVSVGYLVRLVAGHEKVACSGGAPGAGGAGGAGGAAAA   356
                          ********************************************************* **

sp|Q9H461|FZD8_HUMAN      GAGAAGAGAGGPGGRGEYEELGAVEQHVRYETTGPALCTVVFLLVYFFGMASSIWWVILS   418
sp|Q61091|FZD8_MOUSE      GAGAAGAGASSPGARGEYEELGAVEQHVRYETTGPALCTVVFLLVYFFGMASSIWWVILS   416
                          *********..**.**********************************************

sp|Q9H461|FZD8_HUMAN      LTWFLAAGMKWGNEAIAGYSQYFHLAAWLVPSVKSIAVLALSSVDGDPVAGICYVGNQSL   478
sp|Q61091|FZD8_MOUSE      LTWFLAAGMKWGNEAIAGYSQYFHLAAWLVPSVKSIAVLALSSVDGDPVAGICYVGNQSL   476
                          ************************************************************

sp|Q9H461|FZD8_HUMAN      DNLRGFVLAPLVIYLFIGTMFLLAGFVSLFRIRSVIKQQDGPTKTHKLEKLMIRLGLFTV   538
sp|Q61091|FZD8_MOUSE      DNLRGFVLAPLVIYLFIGTMFLLAGFVSLFRIRSVIKQQGGPTKTHKLEKLMIRLGLFTV   536
                          ***************************************.********************

sp|Q9H461|FZD8_HUMAN      LYTVPAAVVVACLFYEQHNRPRWEATHNCPCLRDLQPDQARRPDYAVFMLKYFMCLVVGI   598
sp|Q61091|FZD8_MOUSE      LYTVPAAVVVACLFYEQHNRPRWEATHNCPCLRDLQPDQARRPDYAVFMLKYFMCLVVGI   596
                          ************************************************************

sp|Q9H461|FZD8_HUMAN      TSGVWVWSGKTLESWRSLCTRCCWASKGAAVGGGAGATAAGGGGGPGGGGGGGPGGGGGP   658
sp|Q61091|FZD8_MOUSE      TSGVWVWSGKTLESWRALCTRCCWASKGAAVGAGAGGSG-------PGGSGPGPGGGGGH 649
                          ****************:***************.***.:.        **.* *******

sp|Q9H461|FZD8_HUMAN      GGGGGSLYSDVSTGLTWRSGTASSVSYPKQMPLSQV 694
sp|Q61091|FZD8_MOUSE      GGGGGSLYSDVSTGLTWRSGTASSVSYPKQMPLSQV 685
                          *************************************
```

Figure S4: Sequence alignment of human and murine Fzd8.

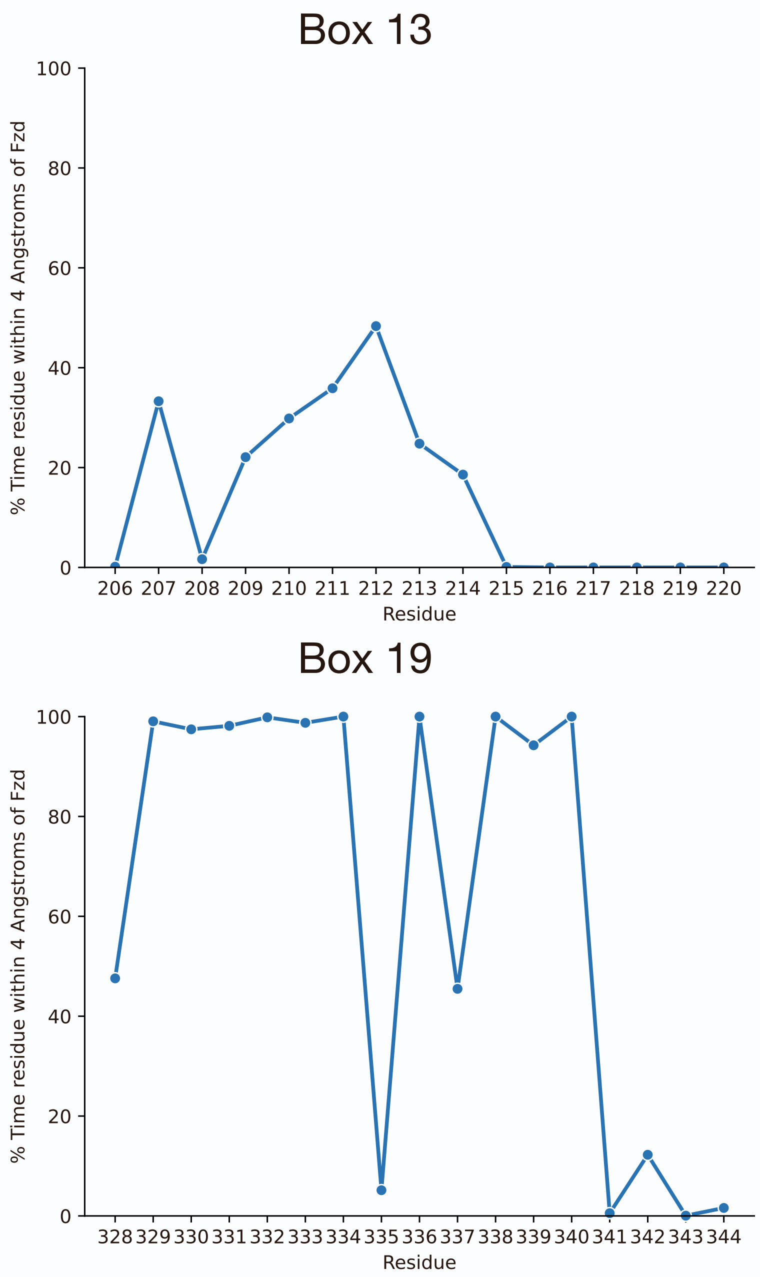

Figure S5: Residence time of the interaction of each amino acid in box 13 and 19 with the Fzd protein. A residue is determined to be in contact with Fzd if it is within 4 Angstroms of any atom in a Fzd residue.

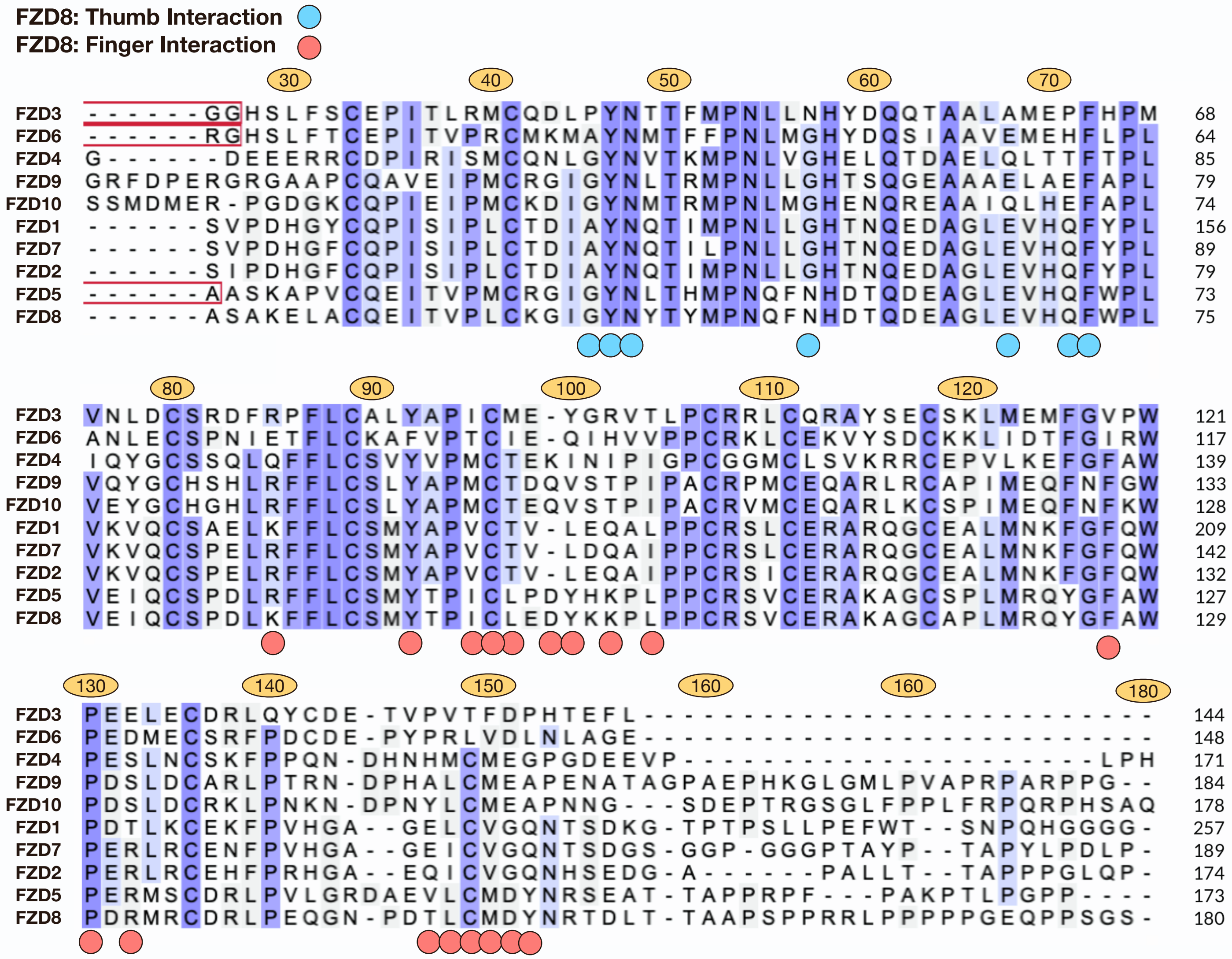

Figure S6: Sequence alignment of all 10 human Fzd protein sequences in the regions interacting with Wnt ligands. Residues interacting (Defined as within 5 angstroms of the Wnt ligand) are highlighted in Blue (Thumb interaction), and Red (Finger interaction).

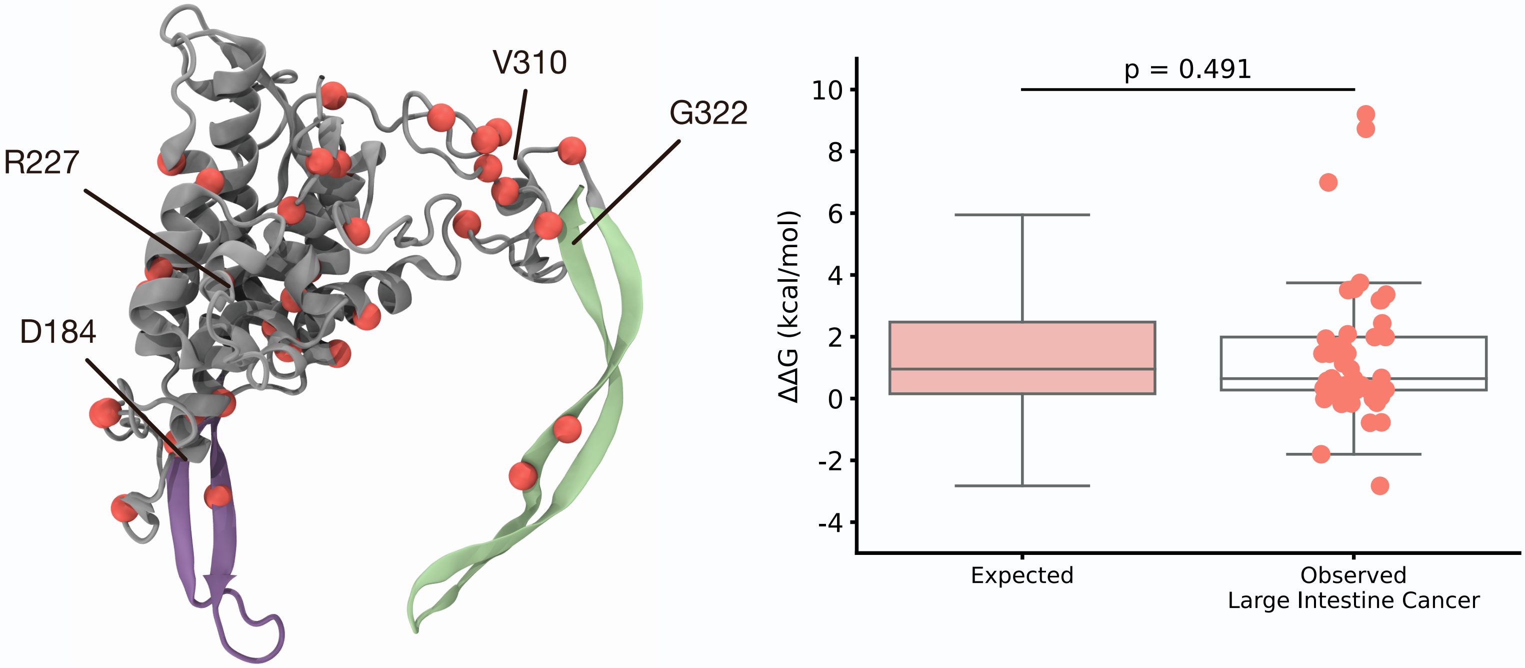

Figure S7. Mutations to Wnt5a found in patients with colorectal adenocarcinoma. (Left) Structure of Wnt5a with mutation sites highlighted as red spheres. (Right) Comparison of folding energy of each mutation (kcal/mol) compared to an expected distribution calculated from the mutational signature. P represents monte-carlo test.

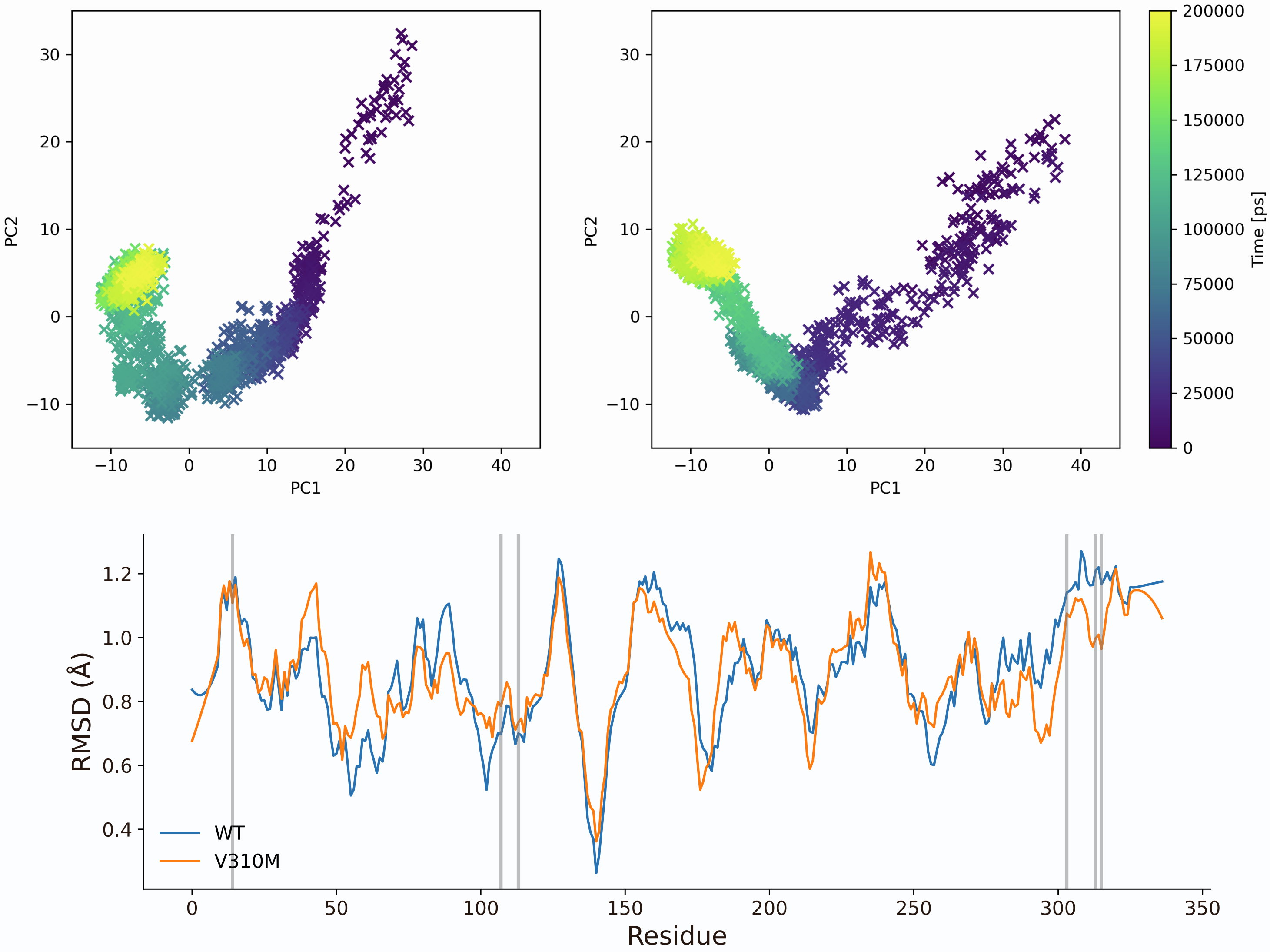

Figure S8: Principal Component Analysis (PCA) for 200ns molecular dynamics simulations for: (top left) WT human WNT5A, (top right) V310M mutated human WNT5A. (Bottom) Root mean square deviation (RMSD) calculations for the trajectories, hinge residues are highlighted as grey lines.
